# Genetic variation of hemolysin co-regulated protein 1 affects the immunogenicity and pathogenicity of *Burkholderia pseudomallei*

**DOI:** 10.1371/journal.pntd.0012758

**Published:** 2025-01-06

**Authors:** Sarunporn Tandhavanant, Thatcha Yimthin, Sineenart Sengyee, Ratana Charoenwattanasatien, Andrey A. Lebedev, Eric R. Lafontaine, Robert J. Hogan, Claire Chewapreecha, T. Eoin West, Paul J. Brett, Mary N. Burtnick, Narisara Chantratita

**Affiliations:** 1 Department of Microbiology and Immunology, Faculty of Tropical Medicine, Mahidol University, Bangkok, Thailand; 2 Department of Bacteriology, Institute of Tropical Medicine, Nagasaki University, Nagasaki, Japan; 3 Department of Microbiology and Immunology, University of Nevada, Reno School of Medicine, Reno, Nevada, United States of America; 4 Beamline Division, Synchrotron Light Research Institute, (Public Organization), Nakhon Ratchasima, Thailand; 5 Center for Biomolecular Structure, Function and Application, Suranaree University of Technology, Nakhon Ratchsima, Thailand; 6 CCP4, Research Complex at Harwell, UKRI–STFC Rutherford Appleton Laboratory, Harwell, Didcot, United Kingdom; 7 Department of Infectious Diseases, College of Veterinary Medicine, University of Georgia, Athens, Georgia, United States of America; 8 Department of Veterinary Biosciences and Diagnostic Imaging, College of Veterinary Medicine, University of Georgia, Athens, Georgia, United States of America; 9 Mahidol-Oxford Tropical Medicine Research Unit, Faculty of Tropical Medicine, Mahidol University, Bangkok, Thailand; 10 Department of Clinical Tropical Medicine, Faculty of Tropical Medicine, Mahidol University, Bangkok, Thailand; 11 Division of Pulmonary, Critical Care and Sleep Medicine, Department of Medicine, University of Washington, Seattle, Washington United States of America; 12 Department of Global Health, University of Washington, Seattle, Washington, United States of America; University of Florida, UNITED STATES OF AMERICA

## Abstract

Hemolysin co-regulated protein 1 (Hcp1) is a component of the cluster 1 Type VI secretion system (T6SS1) that plays a key role during the intracellular lifecycle of *Burkholderia pseudomallei*. Hcp1 is recognized as a promising target antigen for developing melioidosis diagnostics and vaccines. While the gene encoding Hcp1 is retained across *B*. *pseudomallei* strains, variants of *hcp1* have recently been identified. This study aimed to examine the prevalence of *hcp1* variants in clinical isolates of *B*. *pseudomallei*, assess the antigenicity of the Hcp1 variants, and the ability of strains expressing these variants to stimulate multinucleated giant cell (MNGC) formation in comparison to strains expressing wild-type Hcp1 (Hcp1^wt^). Sequence analysis of 1,283 primary clinical isolates of *B*. *pseudomallei* demonstrated the presence of 8 *hcp1* alleles encoding three types of Hcp1 proteins, including Hcp1^wt^ (98.05%), Hcp1^variant A^ (1.87%) and Hcp1^variant B^ (0.08%). Compared to strains expressing Hcp1^wt^, those expressing the dominant variant, Hcp1^variant A^, stimulated lower levels of Hcp1^variant A^-specific antibody responses in melioidosis patients. Interestingly, when Hcp1^variant A^ was expressed in *B*. *pseudomallei* K96243, this strain retained the ability to stimulate MNGC formation in A549 cells. In contrast, however, similar experiments with the Hcp1^variant B^ demonstrated a decreased ability of *B*. *pseudomallei* to stimulate MNGC formation. Collectively, these results show that *B*. *pseudomallei* strains expressing variants of Hcp1 elicit variable antibody responses in melioidosis patients and differ in their ability to promote MNGC formation in cell culture.

## Author summary

Hcp1 is a component of the virulence-associated Type VI Secretion System 1 (T6SS1) that plays a significant role in the MNGC formation of *B*. *pseudomallei* in hosts. In addition to its importance in bacterial pathogenesis, Hcp1 is recognized by the host immune system, resulting in strong antigen-specific humoral and cellular responses in melioidosis patients and protective immunity in animal models. Our analysis of *hcp1* sequences from 1,283 clinical *B*. *pseudomallei* isolates revealed genetic diversity and identified 8 *hcp1* alleles and 3 types of Hcp1. We demonstrated that Hcp1^variant A^, associated with several amino acid changes, could still function in cell-to-cell spread similar to Hcp1^wt^, but it exhibited decreased specific antibody levels in plasma of melioidosis patients compared to the levels stimulated by Hcp1^wt^. Understanding the impact of possible antigenic variation with respect to immune responses and bacterial pathogenesis will be necessary to determine if expression of different Hcp1 proteins may have implications for the development of effective treatments and vaccines.

## Introduction

*Burkholderia pseudomallei* is a Gram-negative bacterium found in the environment in tropical and sub-tropical regions worldwide [1]. It is categorized as a Tier 1 agent by the U.S. Centers for Disease Control and Prevention (CDC) due to its potential for misuse as a biological weapon [2]. *B*. *pseudomallei* can infect humans and a broad range of animals and cause a potentially fatal disease called melioidosis. The bacterium can be contracted by inoculation, inhalation or ingesting contaminated soils and water [3]. Thailand is a hyperendemic area for melioidosis with the highest number of cases being reported in the Northeast region of the country. However, reported cases of melioidosis are underestimated due to misdiagnosis and lack of awareness in several areas [4]. Melioidosis is a life-threatening infectious disease with a high mortality rate of up to 34% and a recurrence rate within one year of up to 2% in Northeast Thailand [5]. The clinical symptoms of melioidosis range from mild, localized infections to severe sepsis. The most common presentations are pneumonia and septic shock with multiple abscesses in internal organs. Currently, no vaccines are available for immunization against melioidosis [6].

*B*. *pseudomallei* is a facultative intracellular bacterium that has high genetic diversity. Several studies have shown that *B*. *pseudomallei* isolates from Southeast Asia are genetically distinct from *B*. *pseudomallei* isolates from other regions [7–14] and that this is likely due to differences in environmental factors as well as human, plant and animal migration patterns [1,3,15,16]. The genetic diversity of *B*. *pseudomallei* strains within the same area can also be high, suggesting that the bacterium has evolved to adapt to local environmental conditions [15–23]. The genetic diversity of *B*. *pseudomallei* has been reported in several virulence factors, including lipopolysaccharide, flagella, BimA, type III secretion system (T3SS) and type VI secretion system (T6SS) [6,11,24]. Of these, variations of LPS and BimA have been shown to be associated with bacterial pathogenicity and immune activation [25–28]. Four types of LPS, including type A, type B, type B2 and rough type, are distributed among *B*. *pseudomallei* populations which exhibit distinct immunogenicity [25,26]. A *B*. *mallei*-like *bimA* sequence variation in some *B*. *pseudomallei* isolates that are commonly found in Australian strains has been reported to be associated with neurological manifestations in melioidosis patients in Australia [27,28].

Understanding genetic diversity and the roles of virulence factors in the pathogenesis of melioidosis is important for developing effective treatments and vaccines against infection by *B*. *pseudomallei*. One critical virulence factor expressed by *B*. *pseudomallei* is the cluster 1 Type VI secretion system (T6SS1) that has been shown to influence the intracellular behavior of this pathogen and be required for multinucleated giant cell (MNGC) formation [29]. Hemolysin co-regulated protein 1 (Hcp1), encoded by *bpss1498* (BPS_RS26895), is a component of T6SS1 and forms the secretion tube of this system that is necessary for the transport of effectors from the bacterial cytoplasm into host cells [29,30]. Hcp1 is an antigen being explored in both serodiagnostic and vaccine development efforts [31–39]. Transcriptome analysis revealed that *hcp1* was not expressed when grown in rich media formulations in the laboratory but is expressed following uptake by host cells [29,31,40]. Importantly, Hcp1-specific antibody responses are detected in patients with acute melioidosis [29–31]. In addition, immunization of mice with vaccine formulations that include Hcp1 results in the production of high-level Hcp1-specific antibody and T-cell responses [38,39,41].

Recently, Hcp1 variation has been reported in a population study of clinical and environmental *B*. *pseudomallei* isolates collected in Ubon Ratchathani, Northeast Thailand. Interestingly, 26% of *B*. *pseudomallei* isolated from a household water supply harbored variant *hcp1*, whereas this variant *hcp1* was present in only 7% of clinical *B*. *pseudomallei* isolates [15]. Genome-wide association studies (GWAS) demonstrated that wild-type *hcp1* was one of 38 disease-associated genes in *B*. *pseudomallei* by both a kmer-based and a pan-genome-based approach [15]. Furthermore, this variant *hcp1* was also discovered in a *B*. *pseudomallei* strain isolated from a soil sample in Ubon Ratchathani that was associated with attenuated virulence in mice [42].

To better understand the presence and distribution of *hcp1* variants in Northeast Thailand, this study aimed to examine a large number of clinical *B*. *pseudomallei* isolates obtained from this region. The prevalence of *hcp1* variants was assessed in 1,283 primary clinical *B*. *pseudomallei* isolates from 9 hospitals in Northeast Thailand, and in addition to wild-type Hcp1 (Hcp1^wt^), two variants designated variant A (Hcp1^variant A^) and variant B (Hcp1^variant B^) were identified. The reactivity of recombinant Hcp1^wt^, Hcp1^variant A^ and Hcp1^variant B^ proteins with plasma from melioidosis patients and healthy donors was assessed. Since Hcp1 is known to play a role in cell-to-cell spread in cell culture [29], the efficiency of multinucleated giant cell (MNGC) formation was determined using *B*. *pseudomallei* strains expressing the three different Hcp1 types. For control purposes, *B*. *pseudomallei* K96243 containing *hcp1*^wt^, *hcp1*^variant A^ or *hcp1*^variant B^ were constructed for use in these assays.

## Materials and methods

### Ethics and biosafety

This study was approved by the Ethics Committee of the Faculty of Tropical Medicine, Mahidol University (MUTM 2015-002-01, MUTM 2018-039-02 and MUTM-EXMPT 2020–005). Written informed consent was obtained from all subjects enrolled in our previous studies [5,35]. Work with live *B*. *pseudomallei* was performed at a biosafety level (BSL-3) following the protocol that was approved by the Institutional Biosafety Committee of the Faculty of Tropical Medicine, Mahidol University (TM-2020-005).

Animal experiments were carried out in strict accordance with the recommendations in the Guide for the Care and Use of Laboratory Animals of the National Institutes of Health. The University of Nevada, Reno Institutional Animal Care and Use Committee (IACUC) or University of Georgia IACUC approved the experiments.

### DNA extraction and whole genome sequencing (WGS) of *B*. *pseudomallei* isolates

*B*. *pseudomallei* isolates were collected from primary clinical specimens obtained from 1,283 melioidosis patients who participated in the DORIM study between July 2015 and December 2018 [5]. The genomic DNA was extracted from *B*. *pseudomallei* using a QIAamp DNA mini kit (Qiagen) [43]. Whole genome sequencing was carried out using a 150-base-read library on the Illumina HiSeq2000 system with a 100-cycle paired-end run. The sequences were assembled and annotated sequences using Velvet v.1.2.10 [44] and obtained from our previous study, which deposited in European Nucleotide Archive (ENA) under study accession number PRJEB25606 and PRJEB35787 [43]. The bacterial strains used in this study are listed in S1 Table.

### Analysis of *hcp1* variants and other T6SS1-related genes

Variations of *hcp1*, *tssB*, *tssC* and *tssE* sequences of clinical *B*. *pseudomallei* isolates were identified from assembly data using the blastn function with default parameters (word size: 11, Match: 2, Mismatch: 3, Existence: 5, Extension: 2) in CLC genomic workbench version 20 (Qiagen). *B*. *pseudomallei* K96243 was used as a wild-type. Nucleic acid sequences of each allele were translated into amino acid sequences using standard genetic code. Representative nucleic acid or amino acid sequences were aligned using CLUSTAL W for multiple alignments [45] in the MEGA 11 [46]. The evolutionary analysis was performed using the Maximum Likelihood method with 1,000 replicates of bootstrap analysis. Tamura-Nei model and Jones-Taylor-Thornton (JTT) models were used for substitution analysis of nucleic acid and amino acid sequences, respectively [47,48]. The *hcp1* variants identified by WGS analysis were verified. We performed Sanger sequencing to confirm the DNA sequences of three variant alleles, including allele 6, allele 7, and allele 8 using c_d_bpss1498-wt_F and c_d_bpss1498-wt_R primer pair (S2 Table). PCR products were purified with ExoSAP-IT (Applied Biosystem) before DNA sequencing.

### Production of recombinant Hcp1 (rHcp1) proteins

*hcp1* genes were amplified from genomic DNA of *B*. *pseudomallei* K96243 for *hcp1*^wt^, DR1235 for *hcp1*^allele 8^ (Hcp1^variant A^) and DR0089 for *hcp1* ^allele 6^ (Hcp1^variant B^) with primers listed in S2 Table. PCR products were cloned into the pGEM-T Easy vector (Promega). *hcp1* genes were subcloned into the NcoI and XhoI restriction sites of expression vector pET28a to produce recombinant Hcp1 proteins (rHcp1^wt^, rHcp1^variant A^ or rHcp1^variant B^) with an N-terminal 6×His-tag. The insert *hcp1* sequences in pGEM-T Easy and pET28a vectors were verified by DNA sequencing. The recombinant Hcp1 expression constructs were transformed into *Escherichia coli* BL21DE (Invitrogen). Expression of the recombinant His-tagged Hcp1 proteins was induced using isopropyl β-D-1-thiogalactopyranoside (IPTG) and proteins were purified by affinity chromatography using HisPur Ni-NTA Resin (Thermo Scientific). Coomassie blue staining of purified rHcp1^wt^, rHcp1^variant A^ and rHcp1^variant B^ via SDS-PAGE showed single bands of protein with molecular weights between 19–21 kDa (S1A Fig). Western blot analysis of these proteins using an anti-His antibody (Sigma; H1029) showed the same banding patterns, indicating that the purified recombinant Hcp1 proteins were of high quality (S1B Fig), suitable for further characterization. For the ELISpot assay, endotoxin was removed from the purified proteins using Pierce high-capacity endotoxin removal resin (Thermo Scientific). Endotoxin levels were determined using a Pierce LAL chromogenic endotoxin Quantitation kit (Thermo Scientific).

### Hcp1 antibody production

To obtain polyclonal antibodies directed against Hcp1^variant A^, purified His-tagged rHcp1^variant A^ (5 μg/dose) formulated in tissue culture-grade PBS (pH 7.2; Gibco) with Alhydrogel 2% (250 μg/dose; Brenntag) and CpG (10 μg/dose; ODN 2006; Invivogen) was administered to female C57BL/6 mice (n = 6 mice per group; Charles River Laboratories) subcutaneously on days 0, 21, and 35. Terminal bleeds were conducted 1 week after the final dose. Polyclonal mouse serum generated against recombinant *Burkholderia mallei* Hcp1 (rHcp1) as part of previous studies was available for use [32,38]. Serum was stored at -80°C until required for use.

The rHcp1-specific monoclonal antibody #3 (mAb-H1-3) was generated by fusing splenocytes obtained from a BALB/c mouse immunized with rHcp1 [32], with Sp2/mIL6 cells (ATCC CRL 2016). The fused cells were plated in methylcellulose medium containing hypoxanthine, aminopterin, and thymidine using a ClonaCell HY kit per the manufacturer’s specifications (Stemcell Technologies). Hybridomas secreting antibodies specific to Hcp1 were identified by ELISA.

### Enzyme-linked immunosorbent assay (ELISA)

ELISA was used to determine IgG antibody levels against rHcp1^wt^, rHcp1^variant A^ and rHcp1^variant B^ in the plasma of melioidosis patients and healthy donors from Northeast Thailand, who were randomly selected from the previous study [5], as previously described [32]. EDTA plasma samples were collected from melioidosis patients on the day bacterial culture results were reported positive for *B*. *pseudomallei* [5]. ELISA was performed in duplicate using 50 μl of 2.5 μg/ml rHcp1^wt^, rHcp1^variant A^ or rHcp1^variant B^ protein. Primary antibodies (human plasma, mAb H1-3, anti-rHcp1 [32,38] or anti-rHcp1^variant A^) and secondary antibodies (anti-human IgG conjugated with a horseradish peroxide (HPR; Dako; P0214) for human antibody detection and anti-mouse immunoglobulins conjugated with HRP (Dako; P0260) for mouse antibody detection) were diluted 2,000-fold before used. Mouse monoclonal anti-polyhistidine antibody (Sigma; H1029) was diluted 3,000-fold before use. A positive result was determined using an OD 450 nm cut-off value of 1.165, as previously evaluated by Receiver Operating Characteristic (ROC) for the diagnosis of melioidosis in the Thai population [32].

### Western blot analysis

Purified rHcp1^wt^, rHcp1^variant A^ or rHcp1^variant B^ was mixed with Laemmli sample buffer at a concentration of 300 μg/ml and heated to 95°C for 10 min. Five microliters of each sample were loaded into 15% acrylamide gel electrophoresis and then transferred to the PVDF membrane by electrotransfer. Membranes were blocked with 5% skim milk for 1 h and then reacted with 1:2,000 dilution of rHcp1-specific mAb H1-3, anti-rHcp1 [32,38] or anti-rHcp1^variant A^ polyclonal in PBS at room temperature for 1 h. The membranes were then probed with a 1:5,000 dilution of HRP-conjugated rabbit anti-mouse antibody (Dako; P0260) in PBS at room temperature for 1 h. The immunoblots were then developed with a 3,3’-diaminobenzidine (DAB) substrate.

### Peripheral blood mononuclear cell (PBMC) isolation

PBMCs were collected from melioidosis patients and healthy donors in Northeast Thailand, randomly selected from the previous study [35]. In brief, PBMCs were isolated from 15 ml of heparinized blood by density gradient centrifugation in Lymphoprep (Axis Shield) using a Sepmate tube (STEMCELL technologies) [35]. Heparinized blood was diluted with an equal volume of RPMI 1640 medium supplemented with 10% fetal bovine serum (FBS) before PBMC isolation. The purified PBMCs were resuspended in FBS supplemented with 10% dimethyl sulfoxide (DMSO) for storage in liquid nitrogen until use.

### IFN-γ ELISpot assays

Cellular immune responses to rHcp1^wt^, rHcp1^variant A^ and rHcp1^variant B^ were measured using IFN- γ ELISpot assays as previously described [35]. Frozen PBMC were rapidly thawed and gently diluted into a pre-warmed RPMI medium containing 10% FBS and Benzonase. The cells were then washed twice with RPMI supplemented with 10% FBS. PBMCs were resuspended in CTL-TEST medium supplemented with 1% L-glutamine and incubated at 37°C with 5% CO_2_ for 1 h before viability was determined. 2 ×10^5^ PBMCs were stimulated with 25 μg/ml of rHcp1^wt^, rHcp1^variant A^ or rHcp1^variant B^ in 96-well plates pre-coated with anti-human IFN-γ antibody at 37°C with 5% CO_2_ for 24 h. Phytohemagglutinin (PHA) was used as a positive control. IFN-γ secretion was detected using a Human IFN-γ single-color enzymatic ELISpot kit (Cellular Technology Ltd.). The ELISpot plates were processed and developed per the manufacturer’s instructions. Plates were imaged using an ImmunoSpot S6 Micro analyzer and IFN-γ-secreting T cells were quantitated using the ImmunoSpot v5.1 professional DC smart count software (Cellular Technology Ltd.). Experiments were performed in duplicate.

### Epitope prediction

The MHC-I and -II binding predictions of Hcp1^wt^, Hcp1^variant A^ and Hcp1^variant B^ were conducted using the Immune Epitope Database (IEDB) analysis resource with NetMHCpan 4.1 EL and NetMHCIIpan 4.1 EL methods [49]. The MHC alleles were selected based on common HLA types in the Thai population, including HLA-A*11:01, -B*46:01, -C*01:02, -DRB1*12:02, -DQA1*01:01 and -DQB1*05:02 [50]. The epitope lengths for MHC-I and -II were set at 9–10 and 15 amino acids, respectively.

### Cell culture and multinucleated giant cell (MNGC) formation assays

MNGC formation induced by *B*. *pseudomallei* was performed using A549 cells essentially as previously described [51]. Briefly, A549 cells at 1.5 × 10^4^ in 96-well plates were infected with *B*. *pseudomallei* at a multiplicity of infection (MOI) 50 and incubated at 37°C with 5% CO_2_ for 2 h. Extracellular bacteria were removed by aspiration and any remaining extracellular bacteria were killed with 250 μg/ml kanamycin. Infected cells were incubated for a further 8 h then fixed with 4% paraformaldehyde in PBS for 30 min and stained with Giemsa as previously described [52]. A MNGC was defined as a single cell with three or more nuclei. The percentage of MNGC formation efficiency was calculated by dividing the number of nuclei within multinucleated cells by the total number of nuclei × 100. The average number of nuclei in a MNGC was determined to represent the MNGC size. Experiments were performed in triplicate and four independent experiments were conducted. The bar graphs show the mean of percentage of MNGC formation efficiency or average number of nuclei from four independent experiments with error bars representing the standard deviation (SD).

### *hcp1* gene expression

One hundred microliters of overnight culture of *B*. *pseudomallei* in LB broth was inoculated into fresh 2 ml of RPMI 1640 medium and then incubated at 37°C with shaking at 200 rpm for 5 h. *hcp1* gene expression was stimulated by 200 μM glutathione (Sigma) for 2 h. One milliliter of bacterial culture was collected for RNA extraction using RNeasy mini kit with RNAprotect Bacteria reagent (Qiagen). DNA contamination was treated by RNase-Free DNase as described by the manufacturer (Qiagen) and verified by no PCR product of *dnaK* gene.

*hcp1* gene expression was determined using iTaq Universal SYBR Green One-Step kit (Bio-rad). Expression of *dnaK* gene was used for normalization (ΔCt) [40]. The normalized *hcp1* gene expression levels were compared between conditions with and without glutathione stimulation (ΔΔCt). Relative gene expression was calculated by 2^-ΔΔCt^. The primers for *hcp1* and *dnaK* genes expression are shown in S2 Table.

### Genetic manipulation of *B*. *pseudomallei*

To generate a *hcp1* deletion mutant, *bpss1498* from *B*. *pseudomallei* strain K96243 was deleted by allelic replacement using established methods [53]. In brief, 219 bp upstream and 462 bp downstream fragments of *hcp1* gene were amplified and ligated together to generate a knockout fragment that was then cloned into pGEM-T Easy vector before being subcloned into pEXKm5 at NotI and EcoRI restriction cut sites. The resulting construct was transferred to *B*. *pseudomallei* K96243 by conjugation with *E*. *coli* RHO3. The mutation by allelic replacement was selected for by sucrose induction at room temperature. The *hcp1* mutant, referred to *B*. *pseudomallei* K96243Δ*hcp1*, was confirmed by PCR and DNA sequencing.

*hcp1*^allele 6^ from *B*. *pseudomallei* DR1235 for Hcp1^variant A^ and *hcp1*^allele 7^ from *B*. *pseudomallei* DR0089 for Hcp1^variant B^ were introduced into *B*. *pseudomallei* K96243Δ*hcp1* by allelic replacement as described above. The insertion fragment was designed by insertion of *hcp1* variants between upstream and downstream of the deletion fragment. The insertion fragments were synthesized by Gene Universal Inc (DE, USA) and cloned into pEXKm5. The insertion vectors were transferred to *B*. *pseudomallei* K96243Δ*hcp1* by conjugation with *E*. *coli* RHO3. The allelic replacement was induced by a culture of the bacteria on a sucrose medium. Two *hcp1* mutants with variant genes referred to *B*. *pseudomallei* K96243Δ*hcp1*::*hcp1*^allele 6^ and *B*. *pseudomallei* K96243Δ*hcp1*::*hcp1*^allele 7^ were confirmed by PCR and DNA sequencing. The primers for the genetic manipulation of *B*. *pseudomallei* are shown in S2 Table.

### Protein crystallization

The initial crystallization and screening of rHcp1^variant B^ was performed using an Oryx8 automated crystallization (Douglas Instrument). The protein concentration was adjusted to 10 mg/ml in PBS pH 7.2 and crystallized using the sitting drop vapor diffusion method across 288 conditions, employing Crystal Screen Cryo (Hampton Research; HR2-133), JCSG-plus (Molecular Dimensions; MD1-37), and Morpheus (Molecular Dimensions; MD1-46) at 18°C. The crystals of the rHcp1^variant B^ were grown in condition consisting of 1.5 M ammonium sulfate, 3.75% v/v 2-propanol, and 25% glycerol. The crystals were frozen in liquid nitrogen for storage and diffraction experiments. The resulting crystals were of high quality and diffracted to 1.58 Å resolution.

### Diffraction data collection and crystal structure analysis

Diffraction data sets were collected at the TPS05a beamline at National Synchrotron Radiation Research Center (NSRRC, Hsinchu, Taiwan) with a Rayonix MX300HS CCD detector at the temperature of 100 K and a wavelength of 0.999840 Å. The diffraction data were processed using autoPROC [54]. Data collection and processing statistics are shown in S3 Table.

All computations were carried out using programs from the CCP4 suite, unless otherwise stated [55,56]. The initial molecular replacement solution was obtained using Phaser and the Hcp1^wt^ monomer (PDB ID 3wx6)[30] as a template. The structure was refined with REFMAC [57] alternating with manual model correction in COOT [58]. Hcp1^variant B^ structure has been deposited with the PDB (PDB ID 8Z7K). The details of the structure analysis are described in S1 Text.

### Statistical analysis

The Wilcoxon matched-pairs signed-ranks test was used to analyze humoral and cellular responses to the various rHcp1 proteins assessed in this study. The Student’s t-test was used to test the differences in MNGC formation efficiency among *B*. *pseudomallei* containing different Hcp1 types, as well as differences in *hcp1* gene expression. ANOVA was performed to evaluate differences in intracellular survival among bacterial strains. *P* ≤ 0.05 was considered a statistically significant difference. The statistical analysis was performed using STATA version 13.0.

## Results

### Variation of *B*. *pseudomallei* Hcp1 in the International Nucleotide Sequence Database Collaboration (INSDC)

**Fig 1:**
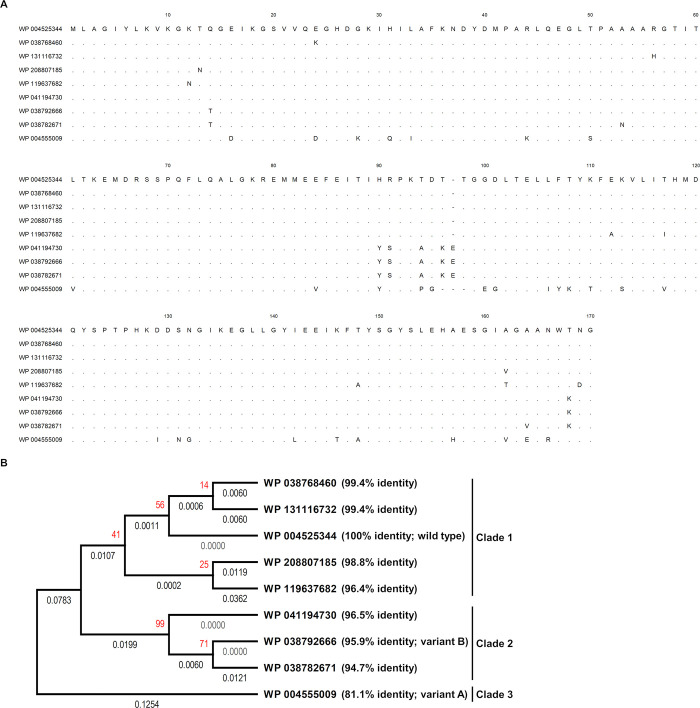
(A) Amino acid alignment and (B) phylogenetic tree based on amino acid sequences of 9 Hcp1 types of *B*. *pseudomallei* recorded in the RefSeq: NCBI Reference Sequence Database on March 4^th^, 2024, generated using the Maximum Likelihood method and JTT matrix-based model with 1,000 bootstraps. Bootstrap values are shown in red text. The branch lengths are presented under each branch.

We initially searched for variations of *B*. *pseudomallei* Hcp1 amino acid sequence that were available in the public database using protein-protein BLAST (blastp) using WP_004525344, identical to Hcp1 of *B*. *pseudomallei* K96243, as query sequence. Nine reference amino acid sequences from RefSeq (NCBI Reference Sequence Database) were found to have 100% coverage with the Hcp1^wt^ and were annotated from *B*. *pseudomallei* (Fig 1A). From the International Nucleotide Sequence Database Collaboration (INSDC), *B*. *pseudomallei* Hcp1 amino acid sequences were annotated for 3,154 records on March 4^th^, 2024. The evolution of Hcp1 was separated into three clades (Fig 1B).

Clade 1 consisted of 5 Hcp1 types, encompassing a total of 2,936 records. The prominent type, WP_004525344, accounted for 2,929 records, found from both clinical and environmental isolates worldwide (2,194 from clinical sources, 629 from environmental sources, 7 from laboratory stock, and 99 from unknown sources). The remaining Hcp1 types in clade 1 included WP_131116732 (2 records from clinical isolates in Sri Lanka), WP_038768460 (1 record from an environmental isolate in Australia), WP_208807185 (2 records from clinical isolates from India), and WP_119637682 (2 records from clinical isolates in USA).

Clade 2 comprised 3 Hcp1 types: WP_041194730 (3 records from 2 environmental isolates in Australia), WP_038792666 (5 records from 3 clinical isolates from Australia, Thailand and the USA, as well as 2 environmental isolates from Australia), and WP_038782671 (1 record from an environmental isolate in Australia).

Interestingly, 6.6% (209/3,154) of the Hcp1 sequences were found in clade 3, represented by WP_004555009 (59 records from clinical sources and 150 records from environmental sources). These were mainly distributed in Thailand and sporadically found in Lao PDR, Vietnam, India and Australia, indicating distinct evolutionary divergence from the other clades.

### Genetic variation of *hcp1* in clinical *B*. *pseudomallei* isolates

We next examined the Hcp1 diversity in clinical isolates of *B*. *pseudomallei* collected from patients admitted at nine different hospitals in Northeast Thailand (S1 Table). In this study, we aimed to examine the variation of *B*. *pseudomallei* Hcp1 by retrieving the full length of *hcp1* gene from the assembly data. Our goal was to identify all known Hcp1 types annotated in a public database and potentially discover new Hcp1 variants. The analysis identified 8 distinct *hcp1* alleles across 1,283 primary clinical isolates (S2 Fig). Among these isolates, the majority (1,208/1,283 isolates, 94.2%) contained a *hcp1* that was identical to that of *B*. *pseudomallei* K96243 (designated as *hcp1*^wt^ or *hcp1*^allele 1^). In 50 *B*. *pseudomallei* isolates (50/1,283 isolates, 3.9%), there were four *hcp1* alleles (*hcp1*^allele 2^, *hcp1*^allele 3^, *hcp1*^allele 4^ and *hcp1*^allele 5^) with more than 99.6–99.8% identity to *hcp1*^wt^, including synonymous single nucleotide polymorphisms (SNPs). The remaining isolates harbored three additional alleles *hcp1*^allele 6^ (*hcp1*^variant B^; N = 1), *hcp1*^allele 7^ (N = 14) and *hcp1*^allele 8^ (N = 10) with identities of 97.5%, 87.1% and 87.3%, respectively, with non-synonymous SNPs compared to *hcp1*^wt^. The *hcp1*^allele 6–8^ were validated by DNA sequencing. Notably, *hcp1*^allele 7^ and *hcp1*^allele 8^ (*hcp1*^variant A^) were on a branch separated from other alleles.

### Amino acid sequence variation of Hcp1 in clinical isolates of *B*. *pseudomallei*

The 8 *hcp1* alleles from clinical isolates of *B*. *pseudomallei* were translated to only three Hcp1 amino acid sequence types (S3 Fig) out of nine types in the INSDC database (Fig 1). The majority of clinical isolates (1,258/1,283 isolates, 98.1%) harbored Hcp1 sequences identical to the reference strain K96243 (WP_004525344) and are representative of wt (allele 1) and alleles 2–5 shown in S2 Fig We identified two other variants of Hcp1 in the 25 remaining *B*. *pseudomallei* isolates. The first Hcp1 variant, designated Hcp1^variant A^ (WP_004555009), was observed in 24 *B*. *pseudomallei* isolates (1.9%). The Hcp1^variant A^ was translated from 2 nucleic acid sequences (*hcp1*^allele 7^ and *hcp1*^allele 8^ in S2 Fig). Compared to Hcp1^wt^, Hcp1^variant A^ had 81.1% amino acid identity (137/169) with two amino acid deletions at positions 96 and 98 in multiple alignments (S3 Fig). Likewise, in *hcp1*^allele 7^ and *hcp1*^allele 8^, the evolutionary analysis demonstrated that Hcp1^variant A^ (WP_004555009) was discrete from other Hcp1 proteins (S3 Fig). The second variant, Hcp1^variant B^ (WP_038792666) was found in only one clinical isolate (DR0089). Compared to Hcp1^wt^, Hcp1^variant B^ showed 95.9% identity (163/170) and harbored an amino acid insertion at position 97 of the alignment (S3 Fig).

Additionally, we found that *B*. *pseudomallei* isolates harboring a Hcp1^variant A^ or Hcp1^variant B^ were dispersed throughout Northeast Thailand. Twenty-five *B*. *pseudomallei* isolates with variants of Hcp1 were cultured from patients admitted to 7 of 9 hospitals in this study. Twenty-four isolates were cultured from Thai patients who resided in 7 provinces in Northeast Thailand and another one isolate was cultured from a Cambodian patient (S1 Table).

### Genomic comparison of *B*. *pseudomallei* isolates with variants of Hcp1

Whole genome sequencing analysis showed that *B*. *pseudomallei* isolates with variant Hcp1 had diverse genetic backgrounds. A total of 223 known sequence types (STs) by multiple locus typing (MLST) analysis were assigned to 1,283 *B*. *pseudomallei* isolates in this study (S1 Table) [43]. Twenty-five *B*. *pseudomallei* isolates with a variant of Hcp1 were assigned to 17 known STs by MLST analysis and were distributed into 13 of 101 lineages by PopPUNK analysis (S1 Table) [43]. Of these, 9 isolates were distributed in major lineages (lineage 1 to 3) [43].

We further analyzed the whole genome sequences for variation in the T6SS1 gene cluster *bpss1493* (BPS_RS26870)–*bpss1511* (BPS_RS26960) of *B*. *pseudomallei* strains that had a variant of Hcp1. Variation in TssC, a T6SS contractile sheath protein encoded by *bpss1497* (BPS_RS26890), was associated with Hcp1 variation. Six amino acid sequence types of TssC were identified in 1,283 *B*. *pseudomallei* isolates in this study (S4 Fig and S4 Table). We observed two conserved amino acid substitutions, V457A and V486I, in 3 types of TssC (TssC^variant C^, TssC^variant D^ and TssC^variant E^) from the 24 *B*. *pseudomallei* isolates that produced Hcp1^variant A^ (S1 Table). These two amino acid substitutions in TssC were not observed in other clinical isolates of *B*. *pseudomallei* in this study. *B*. *pseudomallei* containing *hcp1*^variant B^ contained TssC^wt^, similar to *B*. *pseudomallei* K96243 (S1 Table).

*B*. *pseudomallei* isolates containing *hcp1*^variant A^ were also associated with amino acid substitutions in TssB (a T6SS contractile sheath small subunit) and TssE (a T6SS baseplate subunit) (S1 and S4 Tables and S5 and S6 Figs). Of these 24 isolates, 23 isolates had a conserved amino acid change, T74A, in TssB (TssB^variant D^; S5 Fig). However, the T74A change in TssB that was found in 3 types of TssB, including TssB^variant D^, TssB^variant E^ and TssB^variant F^ was not specific to *B*. *pseudomallei* strains harboring the Hcp1^variant A^. This change was also detected in 165 *B*. *pseudomallei* isolates with Hcp1^wt^ or Hcp1^variant B^. Similar to TssB, 22 *B*. *pseudomallei* isolates containing *hcp1*^variant A^ had conserved amino acid changes, E27A and H32R, in TssE (TssE^variant F^, TssE^variant G^ and TssE^variant I^; S1 Table and S6 Fig) but both non-synonymous SNPs were also detected in 35 *B*. *pseudomallei* isolates with Hcp1^wt^.

### Antigenicity of Hcp1

A recent study investigated the impact of a variant *hcp1* gene found in environmental *B*. *pseudomallei* isolates and its role in virulence in an animal model and found that isolates carrying the variant *hcp1* gene (containing *hcp1*^variant A^; ST93 and ST60) exhibited a reduced level of virulence compared to strains with *hcp1*^wt^ (ST58 and ST176) [42]. It is possible that the different amino acid compositions of Hcp1 proteins may significantly influence their biological properties.

**Fig 2:**
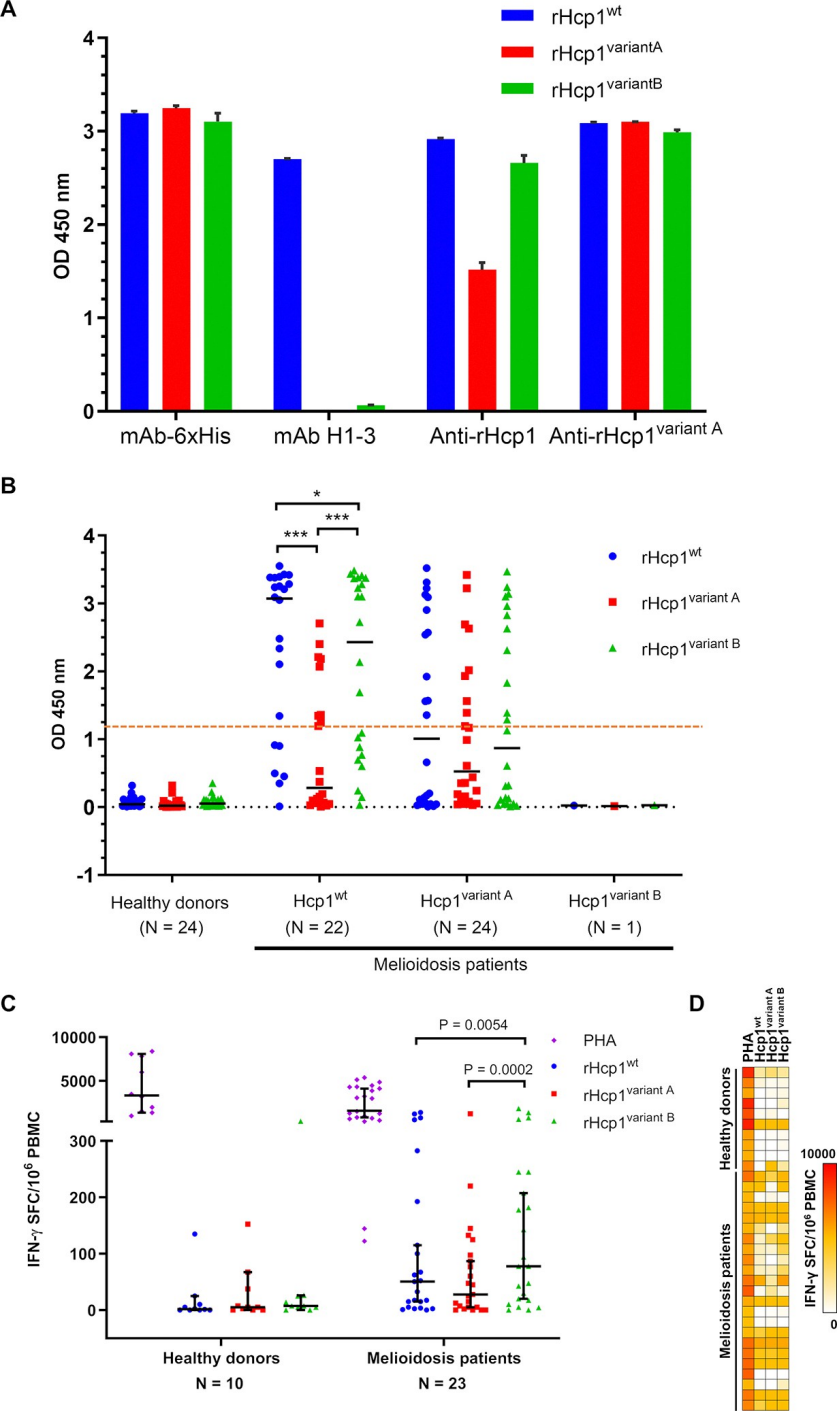
Antigenic variation of recombinant Hcp1 proteins. (A) Reactivity of mouse anti-polyhistidine (mAb-6×His), mouse monoclonal antibody against rHcp1^wt^ (mAb H1-3), mouse sera obtained from *B*. *mallei* rHcp1 immunization (anti-rHcp1) and mouse sera obtained from rHcp1^variant A^ immunization (anti-rHcp1^variant A^) against rHcp1^wt^ (blue bar), rHcp1^variant A^ (red bar) and rHcp1^variant B^ (green bar). The experiment was performed in duplicate. The bar graph represents mean of OD values and error bar shows SD. (B) Reactivity of human sera from healthy donors, melioidosis patients infected with *B*. *pseudomallei* containing *hcp1*^wt^, melioidosis patients infected with *B*. *pseudomallei* containing *hcp1*^variant A^ and melioidosis patient infected with *B*. *pseudomallei* containing *hcp1*^variant B^ against rHcp1^wt^ (blue dot), rHcp1^variant A^ (red square) and rHcp1^variant B^ (green triangle). The data points represent the mean of duplicate OD values. The black line represents the median of each group. The orange dotted line represents the cut-off value. * *P* ≤ 0.05, *** *P* ≤ 0.001. (C) IFN-γ secretion from PBMC of 10 healthy donors and 23 melioidosis patients infected with *B*. *pseudomallei* containing *hcp1*^wt^ after stimulated with PHA (purple diamond), rHcp1^wt^ (blue dot), rHcp1^variant A^ (red square) and rHcp1^variant B^ (green triangle). The data points present the mean of duplicate wells. The error bars represented 95% CI. (D) Heat map of IFN-γ secretion from PBMC of 10 healthy donors and 23 melioidosis patients infected with *B*. *pseudomallei* containing *hcp1*^wt^ after stimulated with PHA, rHcp1^wt^, rHcp1^variant A^ and rHcp1^variant B^. The data points present the mean of duplicate wells.

We predicted the MHC-I and -II binding epitopes of Hcp1^wt^, Hcp1^variant A^ and Hcp1^variant B^ using IEDB analysis resource. The prediction showed 963, 951, and 969 MHC-I binding epitopes for Hcp1^wt^, Hcp1^variant A^, and Hcp1^variant B^, respectively. Additionally, 310, 306, and 312 epitopes were predicted to bind with MHC-II for Hcp1^wt^, Hcp1^variant A^, and Hcp1^variant B^, respectively. We next assessed the cross-reactivity among antibodies against the Hcp1 proteins identified in this study. We performed ELISA and Western blot analyses using three purified rHcp1 proteins, rHcp1^wt^, rHcp1^variant A^ and rHcp1^variant B^. We utilized various antibodies targeting different Hcp1 types, including mouse monoclonal anti-polyhistidine antibody (mAb-6×His), mouse monoclonal antibody specific for Hcp1^wt^ (mAb H1-3), mouse serum raised against *B*. *mallei* rHcp1 by immunization (anti-rHcp1) [32,38] and mouse serum raised against rHcp1^variant A^ by immunization (anti-rHcp1^variant A^). The reactivity of mAb-6×His demonstrated that the binding affinity of three rHcp1 proteins was comparable when coated on the ELISA plate (Fig 2A and S5 Table). Anti-rHcp1 was used for comparison since Hcp1 from *B*. *mallei* and *B*. *pseudomallei* K96243 are >99% identical and have only one amino acid difference [59] at position 148 of alignment in S3 Fig.

We determined the cross-reactivity of the Hcp1 proteins with mAb H1-3 that recognizes the native form of Hcp1^wt^ by ELISA. As shown in Fig 2A, mAb H1-3 reacted with rHcp1^wt^ but not rHcp1^variant A^ or rHcp1^variant B^, suggesting that mAb H1-3 recognized the epitope affected by amino acid substitutions in both variants, which alter the native form of Hcp1^wt^. We next examined the reactivity of rHcp1^wt^, rHcp1^variant A^ and rHcp1^variant B^ with anti-rHcp1 polyclonal serum. Anti-rHcp1 recognizes all three types of rHcp1 with strong reactivity toward rHcp1^wt^ and rHcp1^variant B^ but showed approximately half the reactivity toward rHcp1^variant A^ by ELISA (Fig 2A and S5 Table) and showed only a faint band in Western blot analysis (S7A Fig). Additionally, the decreasing reactivity of anti-rHcp1 with rHcp1^variant A^ was consistently observed in various dilutions of anti-rHcp1 (S8A Fig and S6 Table). Conversely, mice immunized with rHcp1^variant A^ produced antibodies that cross-reacted toward all three Hcp1 proteins at similar levels by both ELISA and Western blot analysis (Figs 2A and S7B). Furthermore, we observed comparable levels of anti-rHcp1^variant A^ activity toward all three types of rHcp1 even when the anti-serum was diluted to 1:10,000 (S8B Fig and S7 Table).

### Antibody responses against Hcp1 variants in melioidosis patients

To assess the impact of Hcp1 variation on the humoral immune responses associated with melioidosis patients, we performed ELISAs to determine the reactivity of IgG antibodies against rHcp1^wt^, rHcp1^variant A^ and rHcp1^variant B^. Plasma samples were collected from healthy donors and three groups of melioidosis patients who were infected with *B*. *pseudomallei* strains containing *hcp1*^wt^, *hcp1*^variant A^ or *hcp1*^variant B^ (Fig 2B and Tables 1 and S8). Plasma samples were obtained from melioidosis patients within 24 h after diagnosis based on the culture method. The median time from admission to blood collection of melioidosis patients infected with *B*. *pseudomallei* containing *hcp1*^wt^ was 4 days (IQR 3–5), which was not significantly different from the patients infected with *B*. *pseudomallei* containing *hcp1*^variant A^, with a median time of 3 days (IQR 2.5–4; P = 0.1890, Mann-Whitney U test). In contrast, blood from the patient infected with *B*. *pseudomallei* containing *hcp1*^variant B^ was collected after 9 days of admission. An OD value of 1.165, previously evaluated by ROC for diagnosis of melioidosis in the Thai population [32], was used as the threshold for positive specific antibody responses.

**Table 1:** Reactivity of plasma from melioidosis patients against the three recombinant *B*. *pseudomallei* Hcp1 proteins.

Types of Hcp1 in infecting strains and number of melioidosis patients	Number of patients and reactivity of IgG antibodies towards 3 rHcp1 proteins^1^					
	rHcp1^wt^		rHcp1^variant A^		rHcp1^variant B^	
	Positive	Negative	Positive	Negative	Positive	Negative
Hcp1^wt^ (N = 22)	16	6	9	13	13	9
Hcp1^variant A^ (N = 24)	12	12	10	14	11	13
Hcp1^variant B^ (N = 1)	0	1	0	1	0	1

^1^ The interpretation of ELISA results for IgG antibody reactivities in the plasma of melioidosis patients against the three recombinant Hcp1 proteins of *B*. *pseudomallei* was determined using a cut-off OD value 1.165 was used to classify plasma samples as positive.

Antibodies from healthy donors had very low reactivity towards rHcp1^wt^, rHcp1^variant A^ and rHcp1^variant B^, with OD 450 values ranging from 0.004–0.352 (Fig 2B and S8 Table). In contrast, melioidosis patients infected with *B*. *pseudomallei* containing *hcp1*^wt^ showed the highest median levels of IgG antibodies specifically targeting rHcp1^wt^, but their antibody levels against rHcp1^variant A^ and rHcp1^variant B^ were lower (P < 0.001 and P = 0.004, respectively; Fig 2B). When an OD 450 value of 1.165 was used as a cut-off to determine a positive result, 16 of 22 (72.7%) melioidosis patients infected with *B*. *pseudomallei* containing *hcp1*^wt^ were detected (Table 1). Among these patients, 9 (56.3%) and 13 (81.3%) of the patients had antibodies that cross-reacted with rHcp1^variant A^ and rHcp1^variant B^ at lower levels (S8 Table). However, 3 of 16 (18.8%) melioidosis cases had negative reactivities with either rHcp1^variant A^ or rHcp1^variant B^ (S8 Table). Only 6 of 22 patients (27.3%) in the melioidosis patient group were infected with *B*. *pseudomallei* strains that produced Hcp1^wt^ and had negative Hcp1-specific IgG levels for all three rHcp1 proteins (S8 Table).

The reactivity of IgG antibodies from melioidosis patients infected with *B*. *pseudomallei* containing *hcp1*^variant A^ showed no significant difference when tested against rHcp1^wt^, rHcp1^variant A^, and rHcp1^variant B^ (rHcp1^wt^ versus and rHcp1^variant A^, P = 0.061; rHcp1^variant A^ versus rHcp1^variant B^, P = 0.077; and rHcp1^wt^ versus rHcp1^variant B^, P = 0.119) (Fig 2B). Reactivity of IgG antibodies against rHcp1^variant A^ was detected in 10 of 24 (41.7%) melioidosis patients infected with *B*. *pseudomallei* containing *hcp1*^variant A^ (S8 Table). Among them, 80.0% (8 of 10) and 70.0% (7 of 10) showed cross-reactivity with rHcp1^wt^ and rHcp1^variant B^ at higher levels than rHcp1^variant A^. Only two melioidosis cases produced specific IgG antibodies that reacted with rHcp1^variant A^ alone. Among 24 melioidosis patients infected with *B*. *pseudomallei* containing *hcp1*^variant A^, 14 (58.3%) were negative for rHcp1^variant A^-specific antibodies. Of these, 4 of 14 melioidosis patients (28.6%) produced antibodies against rHcp1^wt^ and rHcp1^variant B^ but did not react to rHcp1^variant A^. Therefore, 10 of 24 patients (41.7%) in this group had antibody levels against all three recombinant Hcp1 proteins that were below the cut-off value.

The melioidosis patient infected with *B*. *pseudomallei* DR0089 that containing *hcp1*^variant B^ did not have detectable reactivity against purified rHcp1^wt^, rHcp1^variant A^ and rHcp1^variant B^ at the time of enrollment or within 24 h after a positive culture result report.

### Cellular immune responses against variants of Hcp1

To examine cellular immune responses specific for Hcp1^wt^, Hcp1^variant A^ and Hcp1^variant B^, IFN-γ ELISpot assays were conducted. PBMCs isolated from 23 melioidosis patients infected with *B*. *pseudomallei* strains that contained *hcp1*^wt^ were stimulated with rHcp1^wt^, rHcp1^variant A^ or rHcp1^variant B^. PBMCs from 10 healthy donors were used as controls. As expected, PBMCs from both melioidosis patients and healthy donors were responsive to stimulation with PHA, a positive control antigen (S9 Table). Although the purified rHcp1^wt^, rHcp1^variant A^ and rHcp1^variant B^ contained residual endotoxin levels at 8.84, 12.21 and 47.20 EU/ml, respectively, this did not appear to affect the immune response. The healthy donors from Northeast Thailand, an endemic area of melioidosis, may have been previously exposed to *B*. *pseudomallei* during daily life, potentially developing low-level T cell responses to rHcp1 proteins, as previously reported [35]. IFN-γ secretion by PBMCs from healthy donors showed no correlation with endotoxin levels (S9 Table), suggesting that endotoxin contamination did not influence the observed IFN- γ secretion, consistent with previous findings [60, 61]. Moreover, PBMCs from melioidosis patients exhibited IFN-γ-secreting T cell responses when stimulated with all three rHcp1 proteins, with rHcp1^variant B^ inducing significantly IFN-γ secretion than rHcp1^wt^ and rHcp1^variant A^ (P = 0.0054 and 0.0002; Fig 2C and 2D). The rHcp1^variant A^ stimulated IFN-γ secretion from PBMCs at a level similar to that of rHcp1^wt^ (P = 0.330).

### Multinucleated giant cell formation (MNGC) by clinical isolates of *B*. *pseudomallei*

**Fig 3:**
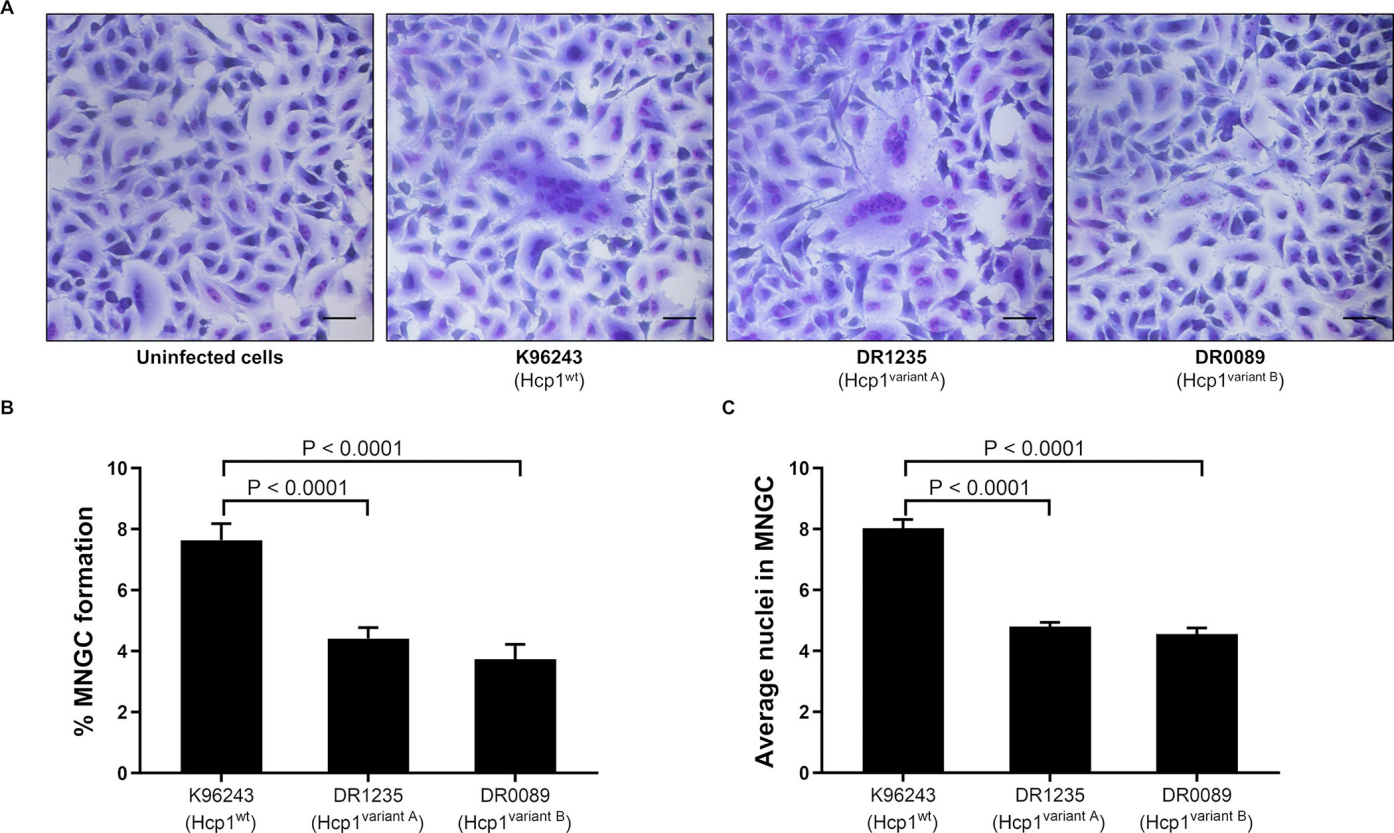
Multinucleated giant cell formation efficiency of clinical *B*. *pseudomallei* isolates with variant *hcp1* genes in A549 cells. (A) Multinucleated giant cell formation in A549 cells infected with strains K96243 (Hcp1^wt^), DR1235 (Hcp1^variant A^) and DR0089 (Hcp1^variant B^) at MOI 50 for 10 h. The bar scale represents 20 μM length. MNGC formation efficiency was calculated by determining the percentage of a MNGC formation (B) and the average number of nuclei in each MNGC (C). The bar graphs show the mean of three independent experiments. The error bars represent the standard deviation (SD).

MNGC formation is a unique phenotype associated with *B*. *pseudomallei* infections, and it is believed that these structures play a role in enabling the bacteria to evade host immune responses [62]. Previous studies have shown that Hcp1 is essential for MNGC formation by *B*. *pseudomallei* in murine macrophages [29]. To investigate a potential role for Hcp1 variation in MNGC formation, A549 cells were infected with *B*. *pseudomallei* at MOI 50 for 10 h and then visualized with Giemsa staining. The clinical *B*. *pseudomallei* isolates producing variant Hcp1 (DR1235 for Hcp1^variant A^ and DR0089 for Hcp1^variant B^) were compared with the reference strain K96243 (produces Hcp1^wt^) for their abilities to stimulate MNGC formation (Fig 3). While the three isolates survived at similar levels in A549 cells (S9A Fig and S10 Table), their ability to stimulate MNGC formation varied (Fig 3A) in both percentage of MNGC formation and average number of nuclei in MNGC (P < 0.001 for all comparisons; Fig 3B and 3C and S11 Table). As expected, *B*. *pseudomallei* K96243 could spread from cell to cell and stimulate the formation of enlarged MNGCs following infection. In contrast, *B*. *pseudomallei* DR1235 induced a lower number of MNGCs with a lower number of nuclei. For *B*. *pseudomallei* DR0089, an expansion of the cytoplasm of the infected cells was observed, but MNGC formation was infrequently detected at 10 h of infection.

### Genomic diversity of *B*. *pseudomallei* influences MNGC formation

It is possible that the MNGC formation associated with clinical isolates of *B*. *pseudomallei* may be influenced by other genes related to intracellular survival and/or transcriptional regulation of T6SS1 genes rather than this being a direct effect of Hcp1 variation. We, therefore, examined the levels of *hcp1* expression by isolates harboring the three different Hcp1 variants. Since glutathione stimulates *hcp1* gene expression *in vitro*, simulating the intracellular phase of infection [63], we determined *hcp1* expression by *B*. *pseudomallei* K93243, DR1235 and DR0089 under glutathione induction. The glutathione-induced expression of *hcp1* in *B*. *pseudomallei* isolates DR1235 (Hcp1^variant A^) and DR0089 (Hcp1^variant B^) was significantly lower than in *B*. *pseudomallei* K96243 (P = 0.023 and 0.010, respectively; S9B Fig and S12 Table). The low *hcp1* expression (S9B Fig) and MNGC formation efficiency (Fig 3B and 3C and S11 Table) suggest that the level of *hcp1* gene expression may influence the efficiency of MNGC formation in A549 cells.

Next, we selected additional clinical isolates of *B*. *pseudomallei* expressing Hcp1^wt^ and Hcp1^variant A^ to compare their abilities to stimulate MNGC formation in A549 cells. Fourteen isolates from each group, representing different multi-locus sequence types (STs), were included for MNGC formation analysis (S13 Table). We observed individual variations in MNGC formation efficiency among clinical *B*. *pseudomallei* isolates. However, the group expressing Hcp1^variant A^ (with 13 STs) showed lower levels of MNGC formation compared to the group expressing Hcp1^wt^ (with 11 STs) (P = 0.027; S9C Fig and S13 Table). However, a group of clinical *B*. *pseudomallei* isolates containing *hcp1*^variant A^ showed varying abilities to form MNGC, suggesting that factors beyond the *hcp1* allele, potentially by the different genetic backgrounds of *B*. *pseudomallei* isolates, may affect gene expression and the ability to stimulate MNGC formation.

### Multinucleated giant cell formation (MNGC) efficiency of *B*. *pseudomallei* K96243 with *hcp1* variants

**Fig 4:**
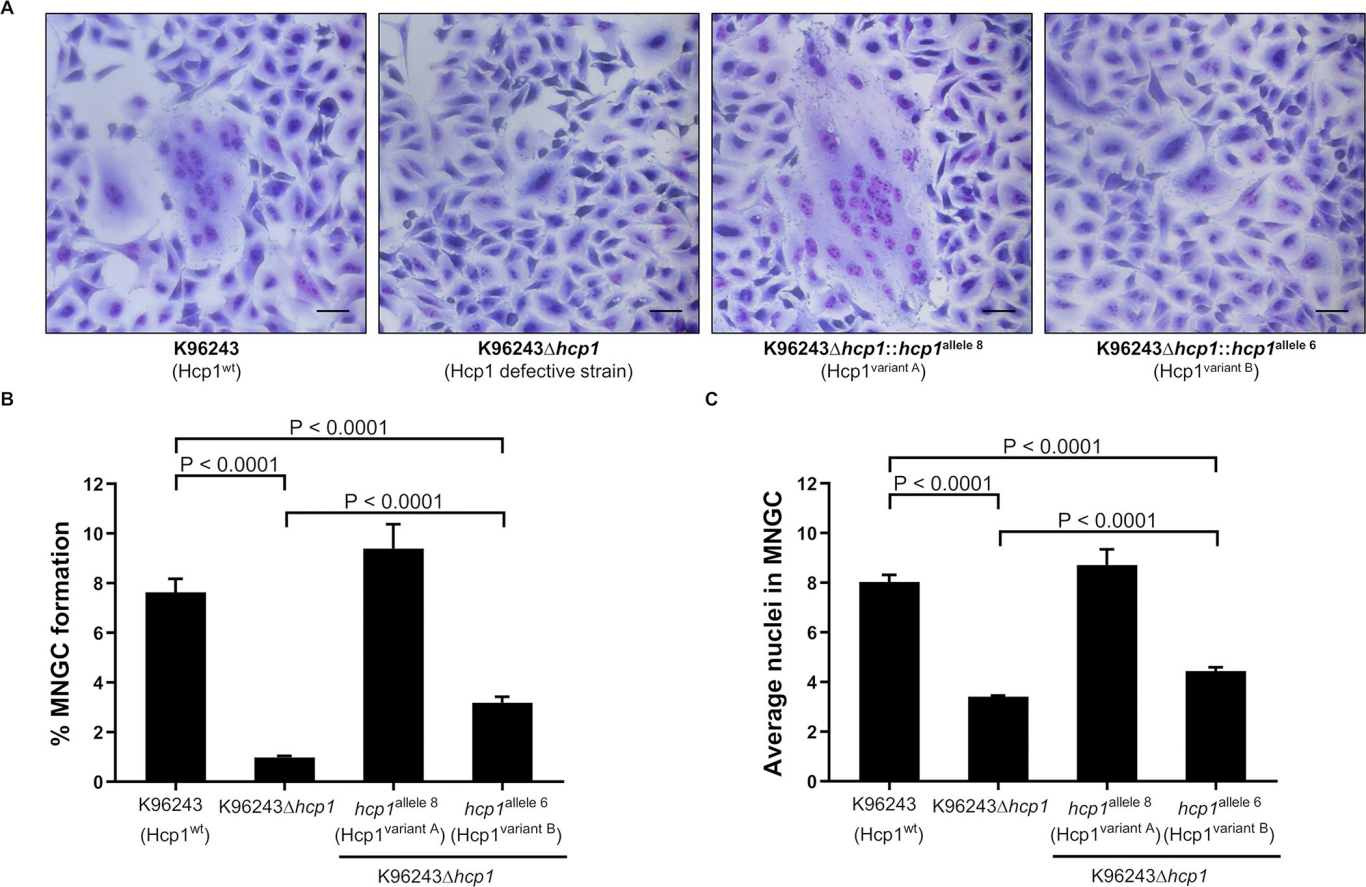
Multinucleated giant cells (MNGC) formation efficiency of genetically manipulated *B*. *pseudomallei* K96243 with variant *hcp1* genes in A549 cells. (A) MNGC of genetically manipulated *B*. *pseudomallei* isolates (strain K96243, K96243Δ*hcp1*, K96243Δ*hcp1*::*hcp1*^allele 6^ and K96243Δ*hcp1*:: *hcp1*^allele 7^) after infection at MOI 50 for 10 h. The bar scale represents 20 μM length. MNGC formation efficiency is presented by the percentage of MNGC formation (B) and average nuclei in MNGC (C). The bar graphs show the mean of three independent experiments. The error bars represent the standard deviation (SD).

Expression of different *hcp1* alleles by clinical isolates of *B*. *pseudomallei* may affect MNGC formation efficiency. To address the possibility that different genetic backgrounds might influence MNGC formation efficiency, we performed mutagenesis experiments to generate derivative strains of *B*. *pseudomallei* K96243 that express Hcp1^variant A^ or Hcp1^variant B^. To facilitate these studies, we first constructed a *hcp1* deletion mutant in *B*. *pseudomallei* K96243 (K96243Δ*hcp1*) and then used this mutant to generate K96243Δ*hcp1*::*hcp1*^allele 8^ (expresses variant A) and K96243Δ*hcp1*::*hcp1*^allele 6^ (expresses variant B). These two strains were then tested for their ability to survive in A549 cells (S10A Fig and S14 Table), levels of *hcp1* gene expression (S10B Fig and S15 Table) and MNGC formation ability (Fig 4) in comparison to K96243 and K96243Δ*hcp1*. All four strains tested, including the wild type K96243, K96243Δ*hcp1*, K96243Δ*hcp1*::*hcp1*^allele 8^ and K96243Δ*hcp1*::*hcp1*^allele 6^, were able to replicate in A549 cells at similar levels (P > 0.05 for all time points; S10A Fig). In addition, *hcp1* expression levels under glutathione stimulation by K96243Δ*hcp1*::*hcp1*^allele 8^ and K96243Δ*hcp1*::*hcp1*^allele 6^ were comparable to wild type K96243 (P > 0.05 for all comparisons; S10B Fig).

Four different *B*. *pseudomallei* strains (K96243, K96243Δ*hcp1*, K96243Δ*hcp1*::*hcp1*^allele 8^ and K96243Δ*hcp1*::*hcp1*^allele 6^) with the same genetic background except for the *hcp1* gene, were evaluated for MNGC formation efficacy after infecting A459 cells for 10 h (Fig 4 and S16 Table). Consistent with previous reports [29,30], we observed that the loss of *hcp1* results in an inability to stimulate MNGC formation, although the bacteria could still survive and multiply within the cells. Expression of the Hcp1^variant A^ in *B*. *pseudomallei* K96243Δ*hcp1*::*hcp1*^allele 8^, restored MNGC formation to levels comparable to the wild-type K96243 (P = 0.131 for % MNGC formation and P = 0.337 for average nuclei in MNGC). In contrast, expression of Hcp1^variant B^ in *B*. *pseudomallei* K96243Δ*hcp1*::*hcp1*^allele 6^ resulted in a slightly increased MNGC formation efficiency compared to K96243Δ*hcp1* (P < 0.0001 for both % MNGC formation and average nuclei in MNGC). However, this strain expressing Hcp1^variant B^ exhibited significantly decreased levels of cell-to-cell spread compared to the wild-type K96243 (P < 0.0001 for both % MNGC formation and average nuclei in MNGC).

### Structural analysis of Hcp1^variant B^

Based on the observation that strains expressing Hcp1^variant B^ did not induce MNGC formation, we hypothesized that this amino acid substitution might affect protein structure and the formation of the tube-like structure of the T6SS1. Therefore, we conducted protein crystallization experiments to examine the structure of Hcp1^variant B^.

Optimized crystallization conditions allowed us to obtain better quality crystals of Hcp1^variant B^ (PDB ID 8z7k, resolution of 1.58 Å) than those used to determine the Hcp1^wt^ structure (PDB ID 3wx6, 2.7 Å) [30]. The new data made it possible: to realize that the crystals are partially disordered and to understand their overall organization, to build and refine a sensible periodic representation of the structure, to re-solve and analyze the structure of 3wx6 and to prove that it is isomorphous to ours, and suffers from the same crystal pathology which was wrongly diagnosed as twinning.

**Fig 5:**
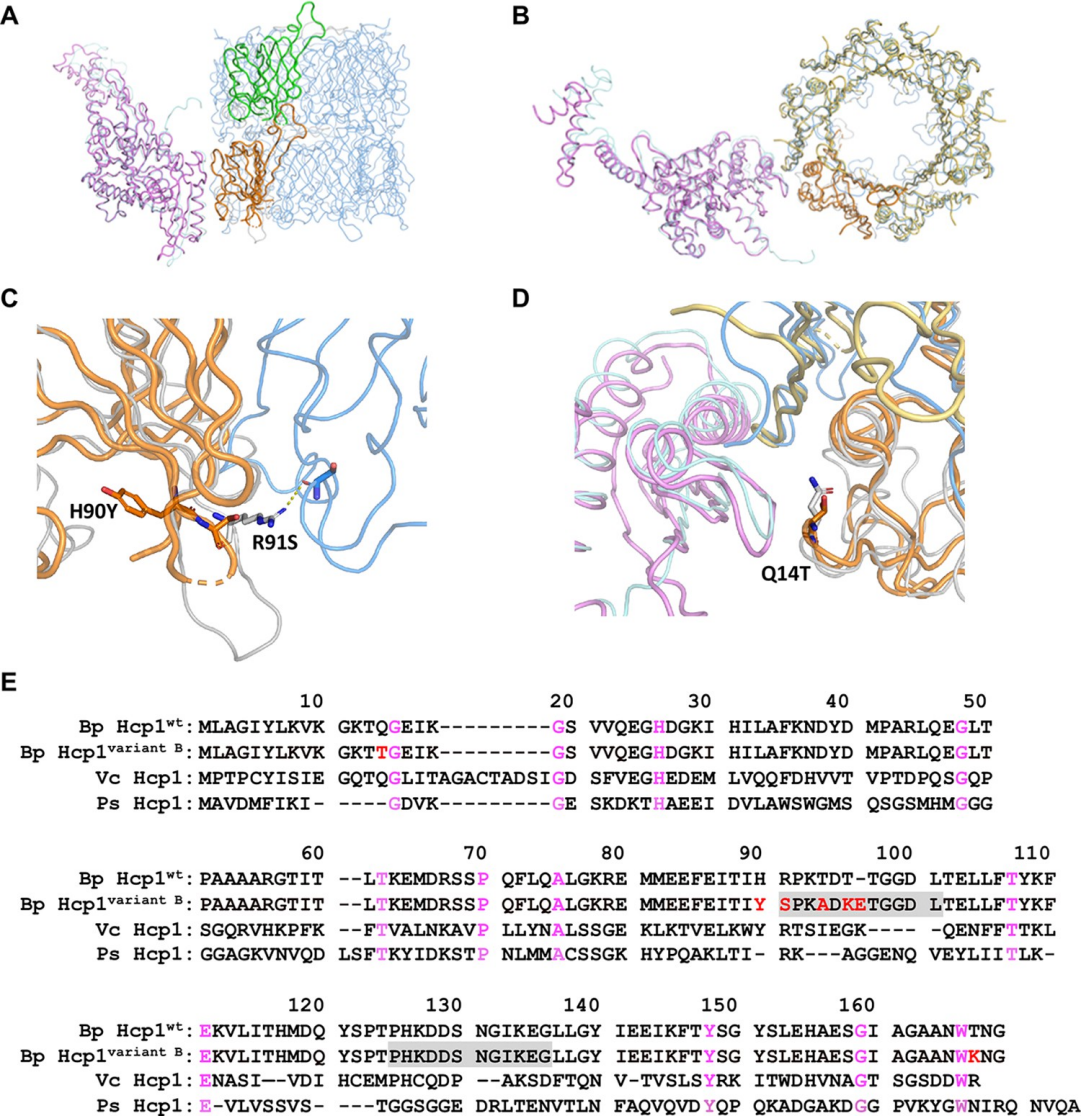
Structural analysis of *B*. *pseudomallei* Hcp1^variant B^. The superposition of the T6SS tubes, comprising Hcp1 and sheath proteins, of *B*. *pseudomallei* and *V*. *cholerae* (5ojq) showed an overall structural similarity and identified intermolecular interfaces that may be impacted by mutations associated with Hcp1^variant B^. X-ray structure of Hcp1^variant B^ (orange, yellow and green) was superposed onto Hcp1^wt^ (3wx6; light blue and grey). AlphaFold2 prediction of *B*. *pseudomallei* sheath protein (magenta) was superposed onto sheath (cyan) proteins in the cryo-EM model of T6SS tubes from *V*. *cholerae* (5ojq). (A) Two superposed subunits of *B*. *pseudomallei* Hcp1^variant B^ (orange and green) highlight a head-to-tail stacking of the Hcp1 hexamers in the T6SS tubes and a twist of the adjacent hexamers. (B) The superposed hexamer of *B*. *pseudomallei* Hcp1^variant B^ (orange and yellow) is formed by symmetry related subunits from the crystal structure and superposition highlights its strong structural conservation. (C) and (D) are zoom-ins of (A) and (B), respectively. (C) Four mutations and an insertion between the residues 90 and 97 are likely to have a strong effect on the conformation of the loops involved in the interactions between hexamers, while (D) the mutation Q14T affects the interaction of the Hcp1 hexamer with the sheath. Mutated residues in (C) and (D) are shown in sticks and the adjacent *V*. *cholerae* Hcp1 subunits in (B) are shown in light blue. (E) The amino acid sequence alignment of *B*. *pseudomallei* Hcp1^wt^ (3wx6) and *B*. *pseudomallei* Hcp1^variant B^ (8z7k) from, *V*. *cholerae* Hcp1 (5ojq) and *P*. *aeruginosa* Hcp1 (1y12). Highlighted regions indicate unmodelled amino acids in Hcp1^variant B^ crystal structure with mutations shown in red and conserved amino acids shown in pink.

Since the 3wx6 structure had been solved, the structure of the T6SS tubes was determined for *Vibrio cholerae* by cryogenic electron microscopy (cryo-EM), PDB ID 5ojq [64]. The structure revealed a head-to-tail stacking of Hcp1 hexamers in the tubes, meaning that the dodecamers observed in both 3xw6 and our structure are crystallographic artifacts. Interestingly, the head-to-tail tubes of Hcp1 had already been observed in a crystal structure of Hcp1 from *Pseudomonas aeruginosa* (1y12) [65] long ago and superposed reasonably well on the tubes reported in the above cryo-EM study, with the main difference being a several-degree twist present in the EM which would not be possible in a crystal structure. The comparison with the above structures allowed us to analyze the possible effect of the mutations on the T6SS tube formation, as illustrated in Fig 5A–5D. Namely, mutations H90Y, R91S, T94A, T96K and T96_T97insE are located either closer to or inside the loop P92-T102 (WT numbering), which is essential for inter-hexamer contacts (Fig 5C), and mutation Q14T is located at the outer surface of the hexamer (Fig 5D) and can be important for the interaction with the sheath proteins. The mutated residues T14, Y90 and S91 are well-defined in our structure (Fig 5C and 5D). Interestingly, Q14 and R91 are conserved in *V*. *cholerae* and *P*. *aeruginosa* (Fig 5E). Additionally, the structure analysis comparing our X-ray structure of Hcp1 with a cryo-EM structure of T6SS sheath/tube complex in *V*. *cholerae* suggested that the amino acid substitution of T167K located near the C-terminus of protein did not affect the structure.

In conclusion, the structural study confirmed the preservation of the hexameric structure in Hcp1^variant B^. This, however, required resolving the 3wx6 structure and verifying that it shared similar crystal pathology with our structure. The possible effect of the mutations on T6SS tube formations in Hcp1^variant B^ was analyzed by comparing our X-ray structure of Hcp1 with a cryo-EM structure of T6SS sheath/tube complex in *V*. *cholerae*. The amino acid substitutions in Hcp1^variant B^ may have a negative effect on T6SS tube formation and, as a result, reduce its effectiveness in MNGC formation.

## Discussion

Several studies have shown that the Hcp1 protein is a promising serodiagnostic marker, therapeutic target, and vaccine candidate for melioidosis [31,33,34,38,39,41]. However, Hcp1 variation has been reported, which may contribute to the virulence of *B*. *pseudomallei* and be associated with pathogenesis of the disease [15,42]. This study identified *hcp1* variants from an extensive collection of 1,283 primary clinical *B*. *pseudomallei* isolates and demonstrated they elicit variable antibody responses in melioidosis patients and can differentially affect MNGC formation in A549 cells. We detected 3 of 9 possible Hcp1 types (WP_004525344, WP_004555009 and WP_038792666) among 1,283 clinical isolates from Northeast Thailand. The other 6 Hcp1 types are rare within the *B*. *pseudomallei* population and may have evolved in other geographically restricted areas. For example, Hcp1 variants in clade 1 were found in environmental isolate from Australia (WP_038768460), clinical isolates from South Asia (WP_131116732 and WP_208807185), and clinical isolates from USA (WP_119637682). Meanwhile, Hcp1 variants in clade 2 (WP_041194730 and WP_038782671) were found in environmental isolates from Australia. This rarity and geographic distribution may explain why these types were not observed in the 1,283 patient samples analyzed in this study. The most common Hcp1 type is identical to that of Hcp1 from *B*. *pseudomallei* K96243 (WP_004525344) and accounted for 98.1% of isolates, which was the predominant Hcp1 type that was annotated in the INDSC.

The major variant designated Hcp1^variant A^ (WP_004555009), which was found in 1.9% (24 of 1,283) from 7 of 9 hospitals, has been reported in previous studies [15,42]. This variant was found in clinical and environmental *B*. *pseudomallei* isolates from Ubon Ratchathani, Northeast Thailand [15, 42]. Additionally, Hcp1^variant A^ was sporadically found in *B*. *pseudomallei* isolates from Laos, Vietnam, India and Australia, annotated in INSDC. A prior population study conducted on *B*. *pseudomallei* isolates from Ubon Ratchathani, Thailand, revealed a higher prevalence of the Hcp1^variant A^ in *B*. *pseudomallei* clinical isolates from Ubon Ratchathani [15], 7.1% (23 of 325), compared to other areas in Northeast Thailand reported in this study. The higher prevalence of Hcp1^variant A^ in water supply isolates in that area [15], 26.6% (114 of 428), might increase the risk of human exposure, leading to a high frequency of clinical Hcp1^variant A^ isolates in Ubon Ratchathani. However, surveillance of *B*. *pseudomallei* in the environment and associated genome analysis has not been conducted in other areas of Northeast Thailand, which is required to gain a better understanding of the distribution of *B*. *pseudomallei* and Hcp1 variation in this region.

The uncommon variant designated Hcp1^variant B^ (WP_038792666) has not been reported in Thailand prior to this study. However, this variant was annotated in INSDC in two environmental isolates from Australia and two clinical isolates from Australia and the USA. The USA patient has a travel history to Australia. These findings suggest that Hcp1^variant B^ may have a broader geographical distribution beyond Thailand, with its presence detected in different regions globally but with limited occurrences.

Genetic variation of *B*. pseudomallei is driven by the selective pressure from environment and infected host [15]. A toxin complex (*tcdBAC*) was associated with clinical isolates whereas a putative adhesin (BPSL1661) has been identified as a co-selection signal for survival under nutrient deprivation [15,16]. Additionally, biofilm formation and the production of 8-O-4′-diferulic acid (a superoxide scavenger metabolite) are essential for persistence within *Acanthamoeba* sp., a phagocytic organism in environments [66]. Antigenic variation is a strategy that some pathogens use to evade the host immune responses [67]. In the case of *B*. *pseudomallei*, genetic diversity in virulence factors such as LPS synthesis and BimA has been associated with bacterial pathogenesis in human [25–28]. The diversity observed in *B*. *pseudomallei* Hcp1 demonstrated in this study may be another mechanism for the bacterium to escape host immune responses, but this will need to be addressed experimentally. At present, it is unclear if the different amino acid compositions in the Hcp1 proteins reported here and associated effects on MNGC formation play a role in the pathogenesis of melioidosis.

Compared with Hcp1^wt^, mutations present in Hcp1^variant A^ were associated with a reduction in the levels of antibody responses to the Hcp1 protein. ELISA results showed that several amino acid substitutions in Hcp1^variant A^ appeared to affect reactivity with Hcp1^wt^-specific mAb H1-3. Additionally, anti-rHcp1 reactivity decreased when reacting with rHcp1^variant A^. Similar to the serum from mice immunized with rHcp1, specific antibodies toward rHcp1^wt^ in plasma of melioidosis patients infected with Hcp1^wt^ isolates had low levels of reactivity with rHcp1^variant A^. This suggests that amino acid substitutions in Hcp1^variant A^ decreased reactivity with Hcp1^wt^-specific antibodies. Therefore, the subunit vaccine developed using Hcp1^wt^ may have reduced efficacy in preventing infection by *B*. *pseudomallei* containing *hcp1*^variant A^.

Interestingly, Hcp1^variant A^ induced antibody responses in mice that recognized all three rHcp1 proteins and did not generate antibody responses that exclusively recognized rHcp1^variant A^. Melioidosis patients infected with Hcp1^variant A^-expressing strains developed antibodies against rHcp1 proteins and most patients had higher antibody levels toward rHcp1^wt^ than rHcp1^variant A^. This correlated with positive ELISA results that showed that melioidosis patients infected with Hcp1^variant A^ isolates were positive less often than those infected with Hcp1^wt^ isolates. It is possible that the amino acid changes may diminish the immunogenicity of Hcp1^variant A^. However, the lower antibody responses to rHcp1 proteins in melioidosis patients infected with *B*. *pseudomallei* with Hcp1^variant A^ may also be due to the host-related factors that may affect immune responses such as underlying diseases and other health conditions.

Despite the amino acid changes associated with the Hcp1^variant A^, strains containing this variant were still capable of inducing MNGC formation. *B*. *pseudomallei* containing *hcp1*^variant A^ and harboring unique TssC types with V457A and V486I, also effectively stimulated MNGC formation in A549 cells. Notably, several *B*. *pseudomallei* isolates containing *hcp1*^variant A^ have been isolated from both environmental and clinical samples, supporting its presence in diverse conditions [15,42].

All 24 *B*. *pseudomallei* isolates containing *hcp1*^variant A^ were carried TssC, a T6SS contractile sheath protein, with amino acid substitutions, V457A and V486I, that found in TssC^variant C^, TssC^variant D^ and TssC^variant E^. Hcp1 and TssC are components of T6SS that have a close interaction. The combination of mutations in these genes might affect the T6SS function. Therefore, an additional experiment is required to verify the effect of a combination of variations of *hcp1* and *tssC* on MNGC formation.

Recently, Roe and colleagues reported that *B*. *pseudomallei* containing *hcp1*^variantA^ from soil isolation are attenuated in mice model of infection comparing with those bacteria contain *hcp1*^wt^ [42]. Additionally, we found that Hcp1 variants had the low antigenicity and *B*. *pseudomallei* containing Hcp1 variants showed low MNGC formation efficiency comparing with Hcp1^wt^. We therefore performed the association analysis between Hcp1 types and 28-day outcomes. Data on 28-day outcomes were available for 1,271 out of 1,283 patients (S17 Table). Among melioidosis patients, 25.5% infected with *B*. *pseudomallei* containing *hcp1*^wt^ and 32.0% infected with *B*. *pseudomallei* containing *hcp1*^variant A^ or *hcp1*^variant B^ died within 28 days after enrollment. The two-by-two table analysis demonstrated that the Hcp1 types were not associated with 28-day outcomes (P = 0.489; Fisher’s exact test), suggesting that the virulence of *B*. *pseudomallei* containing *hcp1*^variant A^ was not significantly different from *B*. *pseudomallei* containing *hcp1*^wt^. However, melioidosis patients present with more complex and uncontrolled variable factors compared to the animal model used by Roe et al [42]. Additionally, virulence gene expression is influenced by the genetic background of each bacterial strain, meaning that *hcp1* variation alone may not be a sole factor affecting patient outcome. The further experiment of animal model infected with *B*. *pseudomallei* strains with the same genetic background but containing different *hcp1* types would provide a clearer demonstration of the impact of *hcp1* variation on *B*. *pseudomallei* virulence.

The genetic diversity of uncommon Hcp1^variant B^ resulted in increased activation of cellular immune responses and decreased MNGC formation. These traits may impose a disadvantage for bacterial infection by *B*. *pseudomallei* containing *hcp1*^variant B^ in an immunocompromised host, as seen in the case of *B*. *pseudomallei* DR0089 infection, which occurred in a patient with immune thrombocytopenic purpura as an underlying condition. Additionally, the limited number of *B*. *pseudomallei* isolates in recorded *B*. *pseudomallei* isolates (only 4) with Hcp1^variant B^ suggests a possible negative selection pressure on its persistence and spread of strains harboring this variant. The combination of increased immune recognition and impaired MNGC formation may contribute to the reduced prevalence of this variant in clinical samples.

Our crystallographic studies showed that rHcp1^variant B^ can form a hexameric ring structure similar to the wild-type protein. The location of mutated residues is in agreement with the possible disruption of inter-hexamer contacts (meaning impaired tube formation) as well as negative effect on the interactions with the sheath proteins. Additional studies would be needed to examine the interactions between each type of Hcp1 protein and other T6SS1 structural proteins, such as TssB, TssC and TssE, to further elucidate the potential role that the Hcp1 variants play in affecting T6SS1 function.

Compared with the prevalence of Hcp1^variant A^ in this study (1.9%), there is a relatively high prevalence (7.1% and 26.6%) from clinical and environmental *B*. *pseudomallei* isolates in Ubon Ratchathani, a hyper-endemic area of melioidosis. While it is possible that the effectiveness of diagnostic tests that rely on detecting Hcp1^wt^ may decrease, as they may fail to detect the Hcp1^variant A^, this is unlikely to have much of an impact given the low percentage of variant isolates seen in clinical samples. Likewise, since there are alternative diagnostic methods available for detection of *B*. *pseudomallei* the existence of Hcp1 variants would not be predicted to impact the diagnosis of melioidosis. Further investigations into the genetic variations and their functional implications will be required to better understand the significance of the different *hcp1* alleles in endemic areas.

### Funding

This research project was supported by Mahidol University to ST. NC and TEW were supported by the US National Institutes of Health U01AI115520. PJB and MNB were supported by Defense Threat Reduction Agency contract HDTRA1-18-C-0062. CC was funded by the Wellcome International Intermediate Fellowship (216457/Z/19/Z) and the Sanger International Fellowship. This research was funded in part by the Wellcome Trust [220211] to NC. For the purpose of Open Access, the author has applied a CC BY public copyright license to any Author Accepted Manuscript version arising from this submission. The funders had no role in study design, data collection and analysis, decision to publish, or preparation of the manuscript.

## Supporting information

S1 Fig Recombinant Hcp1 proteins. Three purified recombinant Hcp1 proteins, including rHcp1^wt^ (Lane1), rHcp1^variant A^ (Lane2) and rHcp1^variant B^ (Lane3) were determined by Coomassie blue stain (A) and Western blot analysis with anti-histidine tag detection (B).
(TIF)

S2 Fig DNA sequence alignment (A) and phylogenetic tree based on DNA sequences (B) of *hcp1*^K96243^ and 7 alleles of *hcp1* presented in 1,283 clinical *B*. *pseudomallei* isolates.
(TIF)

S3 Fig Amino acid sequence alignment (A) and phylogenetic tree based on amino acid sequences (B) of 3 Hcp1 types presented in 1,283 clinical *B*. *pseudomallei* isolates using Hcp1 of *B*. *pseudomallei* K96243 and *B*. *mallei* ATCC23344 as references.
(TIF)

S4 Fig Amino acid sequence alignment (A) and phylogenetic tree based on amino acid sequences (B) of TssC *B*. *pseudomallei* K96243 (wt), and 5 TssC variants (A-E) presented in 1,283 clinical *B*. *pseudomallei* isolates.
(TIF)

S5 Fig Amino acid sequence alignment (A) and phylogenetic tree based on amino acid sequences (B) of TssB *B*. *pseudomallei* K96243 (wt), and 6 TssB variants (A-F) presented in 1,283 clinical *B*. *pseudomallei* isolates.
(TIF)

S6 Fig Amino acid sequence alignment (A) and phylogenetic tree based on amino acid sequences (B) of TssE *B*. *pseudomallei* K96243 (wt), and 9 TssE variants (A-I) presented in 1,283 clinical *B*. *pseudomallei* isolates.
(TIF)

S7 Fig Antigenicity of rHcp1 proteins by western blot analysis. Three purified recombinant Hcp1 proteins, including rHcp1^wt^ (Lane1), rHcp1^variant A^ (Lane2) and rHcp1^variant B^ (Lane3) were blotted on PVDF membrane, reacted to anti-rHcp1 (A) and anti-rHcp1^variant A^ (B), detected with anti-mouse immunoglobulin conjugated with HRP and then visualized by 3,3’-diaminobenzidine.
(TIF)

S8 Fig Reactivity of various dilutions of mouse sera obtained from rHcp1(A) and rHcp1^variant A^ (B) immunizations against rHcp1^wt^ (blue bar), rHcp1^variant A^ (red bar) and rHcp1^variant B^ (green bar). The experiment was performed in duplicate. The bar graph represents mean of OD values and error bar shows SD.
(TIF)

S9 Fig Infection efficiency of clinical *B*. *pseudomallei* isolates. (A) Intracellular replication of *B*. *pseudomallei* in A549 cells after infection at MOI 50. (B) *hcp1* gene expression by *B*. *pseudomallei* after cultured in RPMI1640 medium supplemented with 200 μM glutathione for 2 h. (C) Percentage of MNGC formation by randomly selected clinical *B*. *pseudomallei* isolates.
(TIF)

S10 Fig Infection efficiency of *B*. *pseudomallei* strain K96243 with variant *hcp1* genes. (A) Intracellular replication of *B*. *pseudomallei* in A549 cells after infection at MOI 50. (B) *hcp1* gene expression by *B*. *pseudomallei* after cultured in RPMI1640 medium supplemented with 200 μM glutathione for 2 h.
(TIF)

S1 Table List of clinical *B*. *pseudomallei* isolates in this study. (XLSX)

S2 Table List of primers for recombinant protein productions, genetic manipulation and gene expression. (XLSX)

S3 Table Data collection and refinement statistics of Hcp1^variant B^. (XLSX)

S4 Table Association between Hcp1 types and variations of TssB, TssC and TssE. (XLSX)

S5 Table Reactivity of mAb-6×His, mAb H1-3, anti-rHcp1 and anti-rHcp1^variant A^ against rHcp1^wt^, rHcp1^variant A^ and rHcp1^variant B^. (XLSX)

S6 Table Reactivity of various dilution of anti-rHcp1 against rHcp1^wt^, rHcp1^variant A^ and rHcp1^variant B^. (XLSX)

S7 Table Reactivity of various dilution of anti-rHcp1^variant A^ against rHcp1^wt^, rHcp1^variant A^ and rHcp1^variant B^. (XLSX)

S8 Table Reactivity of human sera from healthy donors, melioidosis patients infected with *B*. *pseudomallei* containing *hcp1*^wt^, melioidosis patients infected with *B*. *pseudomallei* containing *hcp1*^variant A^ and melioidosis patients infected with *B*. *pseudomallei* containing *hcp1*^variant B^ against rHcp1^wt^, rHcp1^variant A^ and rHcp1^variant B^. (XLSX)

S9 Table IFN-γ secretion from PBMC of 10 healthy donors and 23 melioidosis patients infected with *B*. *pseudomallei* containing *hcp1*^wt^ after stimulated with phytohemagglutinin (PHA), rHcp1^wt^, rHcp1^variant A^ and rHcp1^variant B^. (XLSX)

S10 Table Number of intracellular bacteria after A549 cells were infected with *B*. *pseudomallei* K96243, DR1235 and DR0089. (XLSX)

S11 Table Percentage of MNGC formation and average nuclei in MNGC after A549 cells were infected with *B*. *pseudomallei* K96243, DR1235 and DR0089. (XLSX)

S12 Table Fold change of *hcp1* gene expression by *pseudomallei* K96243, DR1235 and DR0089 after exposure to glutathione. (XLSX)

S13 Table List of selected clinical *B*. *pseudomallei* for MNGC formation efficiency determination and percentage of MNGC formation. (XLSX)

S14 Table Number of intracellular bacteria after A549 cells were infected with *B*. *pseudomallei* K96243, K96243Δ*hcp1*, K96243Δ*hcp1*::*hcp1*^allele8^ and K96243Δ*hcp1*::*hcp1*^allele6^. (XLSX)

S15 Table Fold change of *hcp1* gene expression by *pseudomallei* K96243, K96243Δ*hcp1*, K96243Δ*hcp1*::*hcp1*^allele8^ and K96243Δ*hcp1*::*hcp1*^allele6^ after exposure to glutathione. (XLSX)

S16 Table Percentage of MNGC formation and average nuclei in MNGC after A549 cells were infected with *B*. *pseudomallei* K96243, K96243Δ*hcp1*, K96243Δ*hcp1*::*hcp1*^allele8^ and K96243Δ*hcp1*::*hcp1*^allele6^. (XLSX)

S17 Table 28-day outcomes of melioidosis patients infected with *B*. *pseudomallei* containing *hcp1*^wt^, *hcp1*^variantA^, or *hcp1*^variant B^. (XLSX)

S1 Text Diffraction data collection and crystal structure analysis. (PDF)

## References

[1] Currie BJ. Melioidosis and *Burkholderia pseudomallei*: progress in epidemiology, diagnosis, treatment and vaccination. Curr Opin Infect Dis. 2022;35(6):517–23. Epub 20220803. doi: 10.1097/QCO.0000000000000869 . 35942848

[2] Butler D. Viral research faces clampdown. Nature. 2012;490(7421):456. doi: 10.1038/490456a . 23099377

[3] Birnie E, Biemond JJ, Wiersinga WJ. Drivers of melioidosis endemicity: epidemiological transition, zoonosis, and climate change. Curr Opin Infect Dis. 2022;35(3):196–204. doi: 10.1097/QCO.0000000000000827 . 35665713 PMC10128909

[4] Hinjoy S, Hantrakun V, Kongyu S, Kaewrakmuk J, Wangrangsimakul T, Jitsuronk S, et al. Melioidosis in Thailand: present and future. Trop Med Infect Dis. 2018;3(2):38. Epub 2018/05/05. doi: 10.3390/tropicalmed3020038 ; PubMed Central PMCID: PMC5928800. 29725623 PMC5928800

[5] Chantratita N, Phunpang R, Yarasai A, Dulsuk A, Yimthin T, Onofrey LA, et al. Characteristics and one year outcomes of melioidosis patients in Northeastern Thailand: A prospective, multicenter cohort study. Lancet Reg Health Southeast Asia. 2023;9. Epub 20221125. doi: 10.1016/j.lansea.2022.100118 ; PubMed Central PMCID: PMC9788505. 36570973 PMC9788505

[6] Wiersinga WJ, Virk HS, Torres AG, Currie BJ, Peacock SJ, Dance DAB, et al. Melioidosis. Nat Rev Dis Primers. 2018;4:17107. Epub 2018/02/02. doi: 10.1038/nrdp.2017.107 ; PubMed Central PMCID: PMC6456913. 29388572 PMC6456913

[7] Tuanyok A, Auerbach RK, Brettin TS, Bruce DC, Munk AC, Detter JC, et al. A horizontal gene transfer event defines two distinct groups within *Burkholderia pseudomallei* that have dissimilar geographic distributions. J Bacteriol. 2007;189(24):9044–9. Epub 2007/10/16. doi: 10.1128/JB.01264-07 ; PubMed Central PMCID: PMC2168593. 17933898 PMC2168593

[8] Pearson T, Giffard P, Beckstrom-Sternberg S, Auerbach R, Hornstra H, Tuanyok A, et al. Phylogeographic reconstruction of a bacterial species with high levels of lateral gene transfer. BMC Biol. 2009;7:78. Epub 2009/11/20. doi: 10.1186/1741-7007-7-78 ; PubMed Central PMCID: PMC2784454. 19922616 PMC2784454

[9] Price EP, Sarovich DS, Smith EJ, MacHunter B, Harrington G, Theobald V, et al. Unprecedented melioidosis cases in Northern Australia caused by an Asian *Burkholderia pseudomallei* strain identified by using large-scale comparative genomics. Appl Environ Microbiol. 2016;82(3):954–63. Epub 2015/11/27. doi: 10.1128/AEM.03013-15 ; PubMed Central PMCID: PMC4725268. 26607593 PMC4725268

[10] Gee JE, Gulvik CA, Elrod MG, Batra D, Rowe LA, Sheth M, et al. Phylogeography of *Burkholderia pseudomallei* isolates, Western Hemisphere. Emerg Infect Dis. 2017;23(7):1133–8. Epub 2017/06/20. doi: 10.3201/eid2307.161978 ; PubMed Central PMCID: PMC5512505. 28628442 PMC5512505

[11] Chewapreecha C, Holden MT, Vehkala M, Valimaki N, Yang Z, Harris SR, et al. Global and regional dissemination and evolution of *Burkholderia pseudomallei*. Nat Microbiol. 2017;2:16263. Epub 2017/01/24. doi: 10.1038/nmicrobiol.2016.263 ; PubMed Central PMCID: PMC5300093. 28112723 PMC5300093

[12] Gee JE, Gulvik CA, Castelo-Branco D, Sidrim JJC, Rocha MFG, Cordeiro RA, et al. Genomic Diversity of *Burkholderia pseudomallei* in Ceara, Brazil. mSphere. 2021;6(1). Epub 20210203. doi: 10.1128/mSphere.01259-20 ; PubMed Central PMCID: PMC7860993. 33536328 PMC7860993

[13] Jayasinghearachchi HS, Corea EM, Jayaratne KI, Fonseka RA, Muthugama TA, Masakorala J, et al. Biogeography and genetic diversity of clinical isolates of *Burkholderia pseudomallei* in Sri Lanka. PLoS Negl Trop Dis. 2021;15(12):e0009917. Epub 20211201. doi: 10.1371/journal.pntd.0009917 ; PubMed Central PMCID: PMC8824316. 34851950 PMC8824316

[14] Kamthan A, Mukhopadhyay C, Kumar S. Genotyping of *Burkholderia pseudomallei* isolated from patients in south-western coastal region of India. Curr Microbiol. 2022;79(8):226. Epub 20220622. doi: 10.1007/s00284-022-02905-6 . 35731378

[15] Chewapreecha C, Mather AE, Harris SR, Hunt M, Holden MTG, Chaichana C, et al. Genetic variation associated with infection and the environment in the accidental pathogen *Burkholderia pseudomallei*. Commun Biol. 2019;2:428. Epub 2019/12/05. doi: 10.1038/s42003-019-0678-x ; PubMed Central PMCID: PMC6874650. 31799430 PMC6874650

[16] Chewapreecha C, Pensar J, Chattagul S, Pesonen M, Sangphukieo A, Boonklang P, et al. Co-evolutionary signals identify *Burkholderia pseudomallei* survival strategies in a hostile environment. Mol Biol Evol. 2022;39(1). doi: 10.1093/molbev/msab306 ; PubMed Central PMCID: PMC8760936. 34662416 PMC8760936

[17] Chantratita N, Wuthiekanun V, Limmathurotsakul D, Vesaratchavest M, Thanwisai A, Amornchai P, et al. Genetic diversity and microevolution of *Burkholderia pseudomallei* in the environment. PLoS Negl Trop Dis. 2008;2(2):e182. Epub 2008/02/27. doi: 10.1371/journal.pntd.0000182 ; PubMed Central PMCID: PMC2254201. 18299706 PMC2254201

[18] Wuthiekanun V, Limmathurotsakul D, Chantratita N, Feil EJ, Day NP, Peacock SJ. *Burkholderia pseudomallei* is genetically diverse in agricultural land in Northeast Thailand. PLoS Negl Trop Dis. 2009;3(8):e496. Epub 2009/08/05. doi: 10.1371/journal.pntd.0000496 ; PubMed Central PMCID: PMC2713400. 19652701 PMC2713400

[19] McRobb E, Kaestli M, Price EP, Sarovich DS, Mayo M, Warner J, et al. Distribution of *Burkholderia pseudomallei* in northern Australia, a land of diversity. Appl Environ Microbiol. 2014;80(11):3463–8. Epub 2014/03/25. doi: 10.1128/AEM.00128-14 ; PubMed Central PMCID: PMC4018869. 24657869 PMC4018869

[20] Seng R, Saiprom N, Phunpang R, Baltazar CJ, Boontawee S, Thodthasri T, et al. Prevalence and genetic diversity of *Burkholderia pseudomallei* isolates in the environment near a patient’s residence in Northeast Thailand. PLoS Negl Trop Dis. 2019;13(4):e0007348. Epub 2019/04/20. doi: 10.1371/journal.pntd.0007348 . 31002718 PMC6493765

[21] Rachlin A, Mayo M, Webb JR, Kleinecke M, Rigas V, Harrington G, et al. Whole-genome sequencing of *Burkholderia pseudomalle*i from an urban melioidosis hot spot reveals a fine-scale population structure and localised spatial clustering in the environment. Sci Rep. 2020;10(1):5443. Epub 20200325. doi: 10.1038/s41598-020-62300-8 ; PubMed Central PMCID: PMC7096523. 32214186 PMC7096523

[22] Limmathurotsakul D, Holden MT, Coupland P, Price EP, Chantratita N, Wuthiekanun V, et al. Microevolution of *Burkholderia pseudomallei* during an acute infection. J Clin Microbiol. 2014;52(9):3418–21. Epub 2014/06/27. doi: 10.1128/JCM.01219-14 ; PubMed Central PMCID: PMC4313173. 24966357 PMC4313173

[23] Pearson T, Sahl JW, Hepp CM, Handady K, Hornstra H, Vazquez AJ, et al. Pathogen to commensal? Longitudinal within-host population dynamics, evolution, and adaptation during a chronic >16-year *Burkholderia pseudomallei* infection. PLoS Pathog. 2020;16(3):e1008298. Epub 2020/03/07. doi: 10.1371/journal.ppat.1008298 ; PubMed Central PMCID: PMC7077878. 32134991 PMC7077878

[24] Chomkatekaew C, Boonklang P, Sangphukieo A, Chewapreecha C. An evolutionary arms race between *Burkholderia pseudomallei* and host immune system: What do we know? Front Microbiol. 2020;11:612568. Epub 20210121. doi: 10.3389/fmicb.2020.612568 ; PubMed Central PMCID: PMC7858667. 33552023 PMC7858667

[25] Tuanyok A, Stone JK, Mayo M, Kaestli M, Gruendike J, Georgia S, et al. The genetic and molecular basis of O-antigenic diversity in *Burkholderia pseudomallei* lipopolysaccharide. PLoS Negl Trop Dis. 2012;6(1):e1453. Epub 2012/01/12. doi: 10.1371/journal.pntd.0001453 ; PubMed Central PMCID: PMC3250505. 22235357 PMC3250505

[26] Norris MH, Schweizer HP, Tuanyok A. Structural diversity of *Burkholderia pseudomallei* lipopolysaccharides affects innate immune signaling. PLoS Negl Trop Dis. 2017;11(4):e0005571. Epub 2017/04/30. doi: 10.1371/journal.pntd.0005571 ; PubMed Central PMCID: PMC5425228. 28453531 PMC5425228

[27] Morris JL, Fane A, Sarovich DS, Price EP, Rush CM, Govan BL, et al. Increased neurotropic threat from *Burkholderia pseudomallei* strains with a *B*. *mallei*-like variation in the *bimA* motility gene, Australia. Emerg Infect Dis. 2017;23(5):740–9. doi: 10.3201/eid2305.151417 ; PubMed Central PMCID: PMC5403032. 28418830 PMC5403032

[28] Burnard D, Bauer MJ, Falconer C, Gassiep I, Norton RE, Paterson DL, et al. Clinical *Burkholderia pseudomallei* isolates from north Queensland carry diverse *bimABm* genes that are associated with central nervous system disease and are phylogenomically distinct from other Australian strains. PLoS Negl Trop Dis. 2022;16(6):e0009482. Epub 20220614. doi: 10.1371/journal.pntd.0009482 ; PubMed Central PMCID: PMC9236262. 35700198 PMC9236262

[29] Burtnick MN, Brett PJ, Harding SV, Ngugi SA, Ribot WJ, Chantratita N, et al. The cluster 1 type VI secretion system is a major virulence determinant in *Burkholderia pseudomallei*. Infect Immun. 2011;79(4):1512–25. Epub 20110207. doi: 10.1128/IAI.01218-10 ; PubMed Central PMCID: PMC3067527. 21300775 PMC3067527

[30] Lim YT, Jobichen C, Wong J, Limmathurotsakul D, Li S, Chen Y, et al. Extended loop region of Hcp1 is critical for the assembly and function of type VI secretion system in *Burkholderia pseudomallei*. Sci Rep. 2015;5:8235. Epub 2015/02/05. doi: 10.1038/srep08235 ; PubMed Central PMCID: PMC4650826. 25648885 PMC4650826

[31] Chieng S, Mohamed R, Nathan S. Transcriptome analysis of Burkholderia *pseudomalle*i T6SS identifies Hcp1 as a potential serodiagnostic marker. Microb Pathog. 2015;79:47–56. Epub 20150120. doi: 10.1016/j.micpath.2015.01.006 . 25616255

[32] Pumpuang A, Dunachie SJ, Phokrai P, Jenjaroen K, Sintiprungrat K, Boonsilp S, et al. Comparison of O-polysaccharide and hemolysin co-regulated protein as target antigens for serodiagnosis of melioidosis. PLoS Negl Trop Dis. 2017;11(3):e0005499. Epub 2017/03/31. doi: 10.1371/journal.pntd.0005499 ; PubMed Central PMCID: PMC5395236. 28358816 PMC5395236

[33] Phokrai P, Karoonboonyanan W, Thanapattarapairoj N, Promkong C, Dulsuk A, Koosakulnirand S, et al. A rapid immunochromatography test based on Hcp1 is a potential point-of-care test for serological diagnosis of melioidosis. J Clin Microbiol. 2018;56(8). Epub 2018/06/01. doi: 10.1128/JCM.00346-18 ; PubMed Central PMCID: PMC6062804. 29848565 PMC6062804

[34] Tran QTL, Nguyen HV, Pham HT, Mai TV, Nguyen QHM, Le DV, et al. Clinical utility of combined whole-cell antigen and recombinant hemolysis co-regulated protein 1-enzyme-linked immunosorbent assays reveals underdiagnosed cases of melioidosis in Vietnam. Am J Trop Med Hyg. 2022;107(3):585–91. Epub 20220725. doi: 10.4269/ajtmh.21-1143 ; PubMed Central PMCID: PMC9490659. 35895334 PMC9490659

[35] Sengyee S, Yarasai A, Janon R, Morakot C, Ottiwet O, Schmidt LK, et al. Melioidosis patient survival correlates with strong IFN-gamma secreting T cell responses against Hcp1 and TssM. Front Immunol. 2021;12:698303. Epub 20210730. doi: 10.3389/fimmu.2021.698303 ; PubMed Central PMCID: PMC8363298. 34394091 PMC8363298

[36] Whitlock GC, Deeraksa A, Qazi O, Judy BM, Taylor K, Propst KL, et al. Protective response to subunit vaccination against intranasal *Burkholderia mallei* and *B*. *pseudomallei* challenge. Procedia Vaccinol. 2010;2(1). Epub 2010/01/01. doi: 10.1016/j.provac.2010.03.013 ; PubMed Central PMCID: PMC3874274. 24379895 PMC3874274

[37] Muruato LA, Tapia D, Hatcher CL, Kalita M, Brett PJ, Gregory AE, et al. Use of Reverse Vaccinology in the Design and Construction of Nanoglycoconjugate Vaccines against Burkholderia pseudomallei. Clin Vaccine Immunol. 2017;24(11). Epub 20171106. doi: 10.1128/CVI.00206-17 ; PubMed Central PMCID: PMC5674190. 28903988 PMC5674190

[38] Burtnick MN, Shaffer TL, Ross BN, Muruato LA, Sbrana E, DeShazer D, et al. Development of subunit vaccines that provide high-level protection and sterilizing immunity against acute inhalational melioidosis. Infect Immun. 2018;86(1). Epub 2017/11/08. doi: 10.1128/IAI.00724-17 ; PubMed Central PMCID: PMC5736816. 29109172 PMC5736816

[39] Zhu K, Li G, Li J, Zheng M, Peng X, Rao Y, et al. Hcp1-loaded staphylococcal membrane vesicle vaccine protects against acute melioidosis. Front Immunol. 2022;13:1089225. Epub 20221223. doi: 10.3389/fimmu.2022.1089225 ; PubMed Central PMCID: PMC9822774. 36618368 PMC9822774

[40] Chen Y, Wong J, Sun GW, Liu Y, Tan GY, Gan YH. Regulation of type VI secretion system during *Burkholderia pseudomallei* infection. Infect Immun. 2011;79(8):3064–73. Epub 20110613. doi: 10.1128/IAI.05148-11 ; PubMed Central PMCID: PMC3147588. 21670170 PMC3147588

[41] Klimko CP, Shoe JL, Rill NO, Hunter M, Dankmeyer JL, Talyansky Y, et al. Layered and integrated medical countermeasures against *Burkholderia pseudomallei* infections in C57BL/6 mice. Front Microbiol. 2022;13:965572. Epub 20220817. doi: 10.3389/fmicb.2022.965572 ; PubMed Central PMCID: PMC9432870. 36060756 PMC9432870

[42] Roe C, Vazquez AJ, Phillips PD, Allender CJ, Bowen RA, Nottingham RD, et al. Multiple phylogenetically-diverse, differentially-virulent *Burkholderia pseudomallei* isolated from a single soil sample collected in Thailand. PLoS Negl Trop Dis. 2022;16(2):e0010172. Epub 20220210. doi: 10.1371/journal.pntd.0010172 ; PubMed Central PMCID: PMC8865643. 35143500 PMC8865643

[43] Seng R, Chomkatekaew C, Tandhavanant S, Saiprom N, Phunpang R, Thaipadungpanit J, et al. Genetic diversity, determinants, and dissemination of *Burkholderia pseudomallei* lineages implicated in melioidosis in Northeast Thailand. Nat Commun. 2024;15(1):5699. Epub 20240707. doi: 10.1038/s41467-024-50067-9 ; PubMed Central PMCID: PMC11228029. 38972886 PMC11228029

[44] Zerbino DR, Birney E. Velvet: algorithms for de novo short read assembly using de Bruijn graphs. Genome Res. 2008;18(5):821–9. Epub 20080318. doi: 10.1101/gr.074492.107 ; PubMed Central PMCID: PMC2336801. 18349386 PMC2336801

[45] Thompson JD, Higgins DG, Gibson TJ. CLUSTAL W: improving the sensitivity of progressive multiple sequence alignment through sequence weighting, position-specific gap penalties and weight matrix choice. Nucleic Acids Res. 1994;22(22):4673–80. doi: 10.1093/nar/22.22.4673 ; PubMed Central PMCID: PMC308517. 7984417 PMC308517

[46] Tamura K, Stecher G, Kumar S. MEGA11: Molecular Evolutionary Genetics Analysis Version 11. Mol Biol Evol. 2021;38(7):3022–7. doi: 10.1093/molbev/msab120 ; PubMed Central PMCID: PMC8233496. 33892491 PMC8233496

[47] Tamura K, Nei M. Estimation of the number of nucleotide substitutions in the control region of mitochondrial DNA in humans and chimpanzees. Mol Biol Evol. 1993;10(3):512–26. doi: 10.1093/oxfordjournals.molbev.a040023 . 8336541

[48] Jones DT, Taylor WR, Thornton JM. The rapid generation of mutation data matrices from protein sequences. Comput Appl Biosci. 1992;8(3):275–82. doi: 10.1093/bioinformatics/8.3.275 . 1633570

[49] Kaabinejadian S, Barra C, Alvarez B, Yari H, Hildebrand WH, Nielsen M. Accurate MHC Motif Deconvolution of Immunopeptidomics Data Reveals a Significant Contribution of DRB3, 4 and 5 to the Total DR Immunopeptidome. Front Immunol. 2022;13:835454. Epub 20220126. doi: 10.3389/fimmu.2022.835454 ; PubMed Central PMCID: PMC8826445. 35154160 PMC8826445

[50] Satapornpong P, Jinda P, Jantararoungtong T, Koomdee N, Chaichan C, Pratoomwun J, et al. Genetic Diversity of HLA Class I and Class II Alleles in Thai Populations: Contribution to Genotype-Guided Therapeutics. Front Pharmacol. 2020;11:78. Epub 20200227. doi: 10.3389/fphar.2020.00078 ; PubMed Central PMCID: PMC7057685. 32180714 PMC7057685

[51] Sangsri T, Saiprom N, Tubsuwan A, Monk P, Partridge LJ, Chantratita N. Tetraspanins are involved in *Burkholderia pseudomallei*-induced cell-to-cell fusion of phagocytic and non-phagocytic cells. Sci Rep. 2020;10(1):17972. Epub 20201021. doi: 10.1038/s41598-020-74737-y ; PubMed Central PMCID: PMC7577983. 33087788 PMC7577983

[52] Kespichayawattana W, Rattanachetkul S, Wanun T, Utaisincharoen P, Sirisinha S. *Burkholderia pseudomallei* induces cell fusion and actin-associated membrane protrusion: a possible mechanism for cell-to-cell spreading. Infect Immun. 2000;68(9):5377–84. Epub 2000/08/19. doi: 10.1128/iai.68.9.5377–5384.2000 ; PubMed Central PMCID: PMC101801. 10948167 PMC101801

[53] Lopez CM, Rholl DA, Trunck LA, Schweizer HP. Versatile dual-technology system for markerless allele replacement in *Burkholderia pseudomallei*. Appl Environ Microbiol. 2009;75(20):6496–503. Epub 2009/08/25. doi: 10.1128/AEM.01669-09 ; PubMed Central PMCID: PMC2765137. 19700544 PMC2765137

[54] Vonrhein C, Flensburg C, Keller P, Sharff A, Smart O, Paciorek W, et al. Data processing and analysis with the autoPROC toolbox. Acta Crystallogr D Biol Crystallogr. 2011;67(Pt 4):293–302. Epub 20110318. doi: 10.1107/S0907444911007773 ; PubMed Central PMCID: PMC3069744. 21460447 PMC3069744

[55] Winn MD, Ballard CC, Cowtan KD, Dodson EJ, Emsley P, Evans PR, et al. Overview of the CCP4 suite and current developments. Acta Crystallogr D Biol Crystallogr. 2011;67(Pt 4):235–42. Epub 20110318. doi: 10.1107/S0907444910045749 ; PubMed Central PMCID: PMC3069738. 21460441 PMC3069738

[56] Potterton L, Agirre J, Ballard C, Cowtan K, Dodson E, Evans PR, et al. CCP4i2: the new graphical user interface to the CCP4 program suite. Acta Crystallogr D Struct Biol. 2018;74(Pt 2):68–84. Epub 20180201. doi: 10.1107/S2059798317016035 ; PubMed Central PMCID: PMC5947771. 29533233 PMC5947771

[57] Murshudov GN, Skubak P, Lebedev AA, Pannu NS, Steiner RA, Nicholls RA, et al. REFMAC5 for the refinement of macromolecular crystal structures. Acta Crystallogr D Biol Crystallogr. 2011;67(Pt 4):355–67. Epub 20110318. doi: 10.1107/S0907444911001314 ; PubMed Central PMCID: PMC3069751. 21460454 PMC3069751

[58] Emsley P, Cowtan K. Coot: model-building tools for molecular graphics. Acta Crystallogr D Biol Crystallogr. 2004;60(Pt 12 Pt 1):2126–32. Epub 20041126. doi: 10.1107/S0907444904019158 . 15572765

[59] Schell MA, Ulrich RL, Ribot WJ, Brueggemann EE, Hines HB, Chen D, et al. Type VI secretion is a major virulence determinant in *Burkholderia mallei*. Mol Microbiol. 2007;64(6):1466–85. doi: 10.1111/j.1365-2958.2007.05734.x . 17555434

[60] De Groote D, Zangerle PF, Gevaert Y, Fassotte MF, Beguin Y, Noizat-Pirenne F, et al. Direct stimulation of cytokines (IL-1 beta, TNF-alpha, IL-6, IL-2, IFN-gamma and GM-CSF) in whole blood. I. Comparison with isolated PBMC stimulation. Cytokine. 1992;4(3):239–48. doi: 10.1016/1043-4666(92)90062-v . 1498259

[61] Jansky L, Reymanova P, Kopecky J. Dynamics of cytokine production in human peripheral blood mononuclear cells stimulated by LPS or infected by *Borrelia*. Physiol Res. 2003;52(5):593–8. . 14535835

[62] Lennings J, West TE, Schwarz S. The *Burkholderia* Type VI Secretion System 5: Composition, Regulation and Role in Virulence. Front Microbiol. 2018;9:3339. Epub 20190110. doi: 10.3389/fmicb.2018.03339 ; PubMed Central PMCID: PMC6335564. 30687298 PMC6335564

[63] Wong J, Chen Y, Gan YH. Host cytosolic glutathione sensing by a membrane histidine kinase activates the type VI secretion system in an intracellular bacterium. Cell Host Microbe. 2015;18(1):38–48. Epub 2015/06/23. doi: 10.1016/j.chom.2015.06.002 . 26094804

[64] Wang J, Brackmann M, Castano-Diez D, Kudryashev M, Goldie KN, Maier T, et al. Cryo-EM structure of the extended type VI secretion system sheath-tube complex. Nat Microbiol. 2017;2(11):1507–12. Epub 20170925. doi: 10.1038/s41564-017-0020-7 . 28947741

[65] Mougous JD, Cuff ME, Raunser S, Shen A, Zhou M, Gifford CA, et al. A virulence locus of *Pseudomonas aeruginosa* encodes a protein secretion apparatus. Science. 2006;312(5779):1526–30. doi: 10.1126/science.1128393 ; PubMed Central PMCID: PMC2800167. 16763151 PMC2800167

[66] Bunma C, Noinarin P, Phetcharaburanin J, Chareonsudjai S. Burkholderia pseudomallei biofilm resists Acanthamoeba sp. grazing and produces 8-O-4’-diferulic acid, a superoxide scavenging metabolite after passage through the amoeba. Sci Rep. 2023;13(1):16578. Epub 20231003. doi: 10.1038/s41598-023-43824-1 ; PubMed Central PMCID: PMC10547685. 37789212 PMC10547685

[67] Deitsch KW, Lukehart SA, Stringer JR. Common strategies for antigenic variation by bacterial, fungal and protozoan pathogens. Nat Rev Microbiol. 2009;7(7):493–503. Epub 20090608. doi: 10.1038/nrmicro2145 ; PubMed Central PMCID: PMC3676878. 19503065 PMC3676878

