# Supplementary figures and images for: Genetic variation of hemolysin co-regulated protein 1 affects the immunogenicity and pathogenicity of Burkholderia pseudomallei
Source: PLoS Negl Trop Dis. 2025 Jan 6;19(1):e0012758. doi: 10.1371/journal.pntd.0012758 (PMC11737846; doi:10.1371/journal.pntd.0012758)

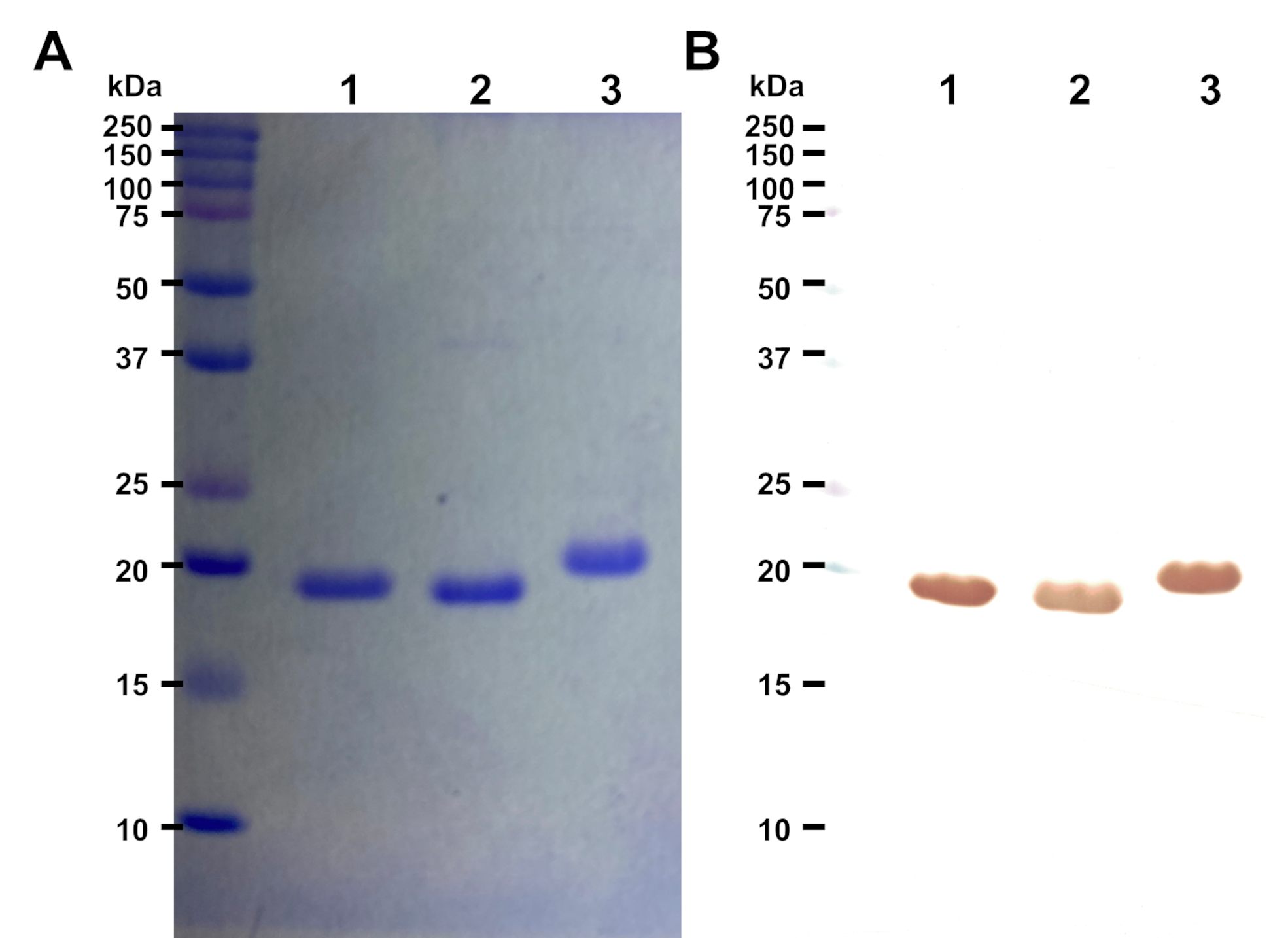

Supplement: S1 Fig — Three purified recombinant Hcp1 proteins, including rHcp1wt (Lane1), rHcp1variant A (Lane2) and rHcp1variant B (Lane3) were determined by Coomassie blue stain (A) and Western blot analysis with anti-histidine tag detection (B). (TIF) [file pntd.0012758.s001.tif]

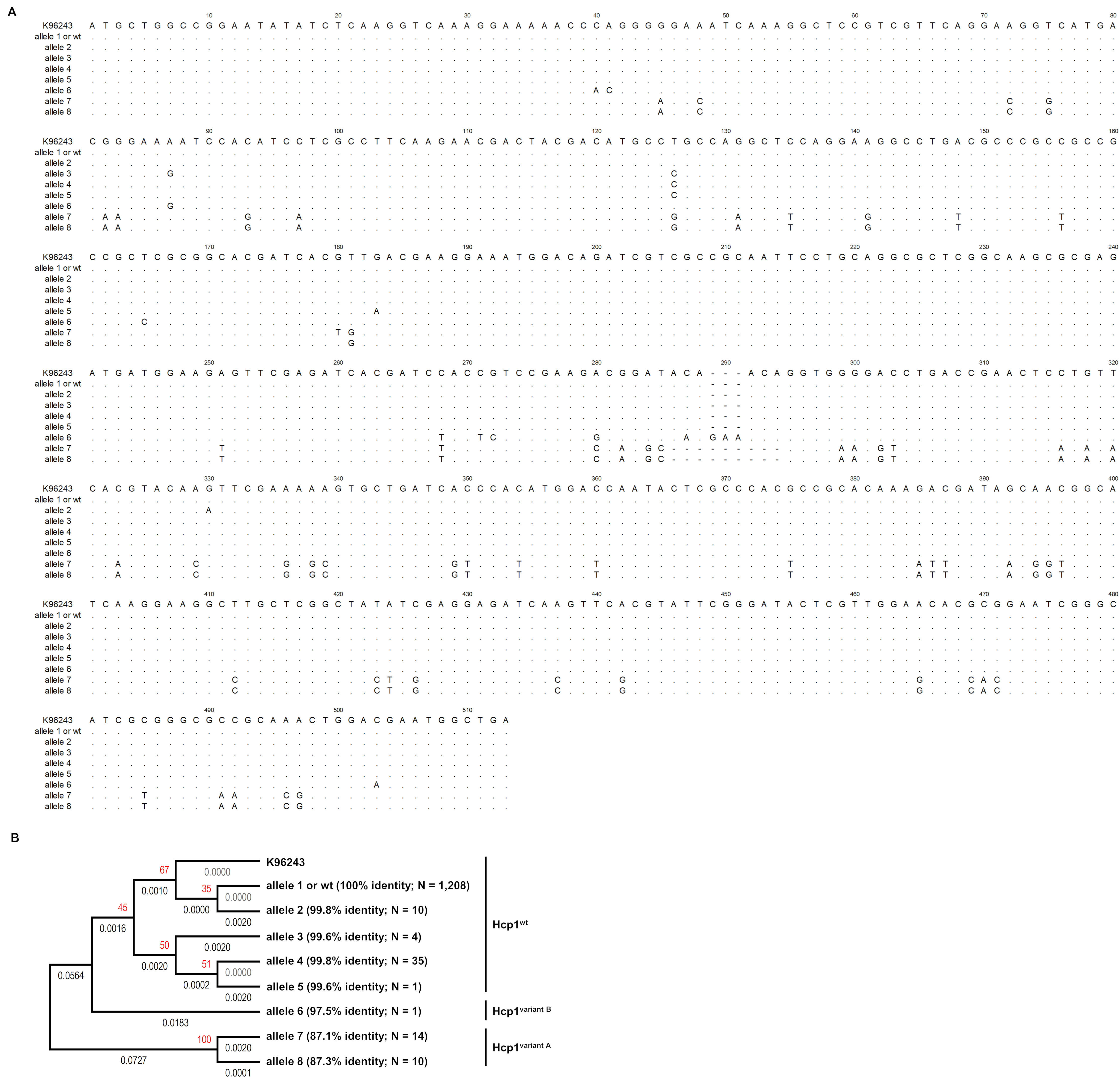

Supplement: S2 Fig — DNA sequence alignment (A) and phylogenetic tree based on DNA sequences (B) of hcp1K96243 and 7 alleles of hcp1 presented in 1,283 clinical B. pseudomallei isolates. (TIF) [file pntd.0012758.s002.tif]

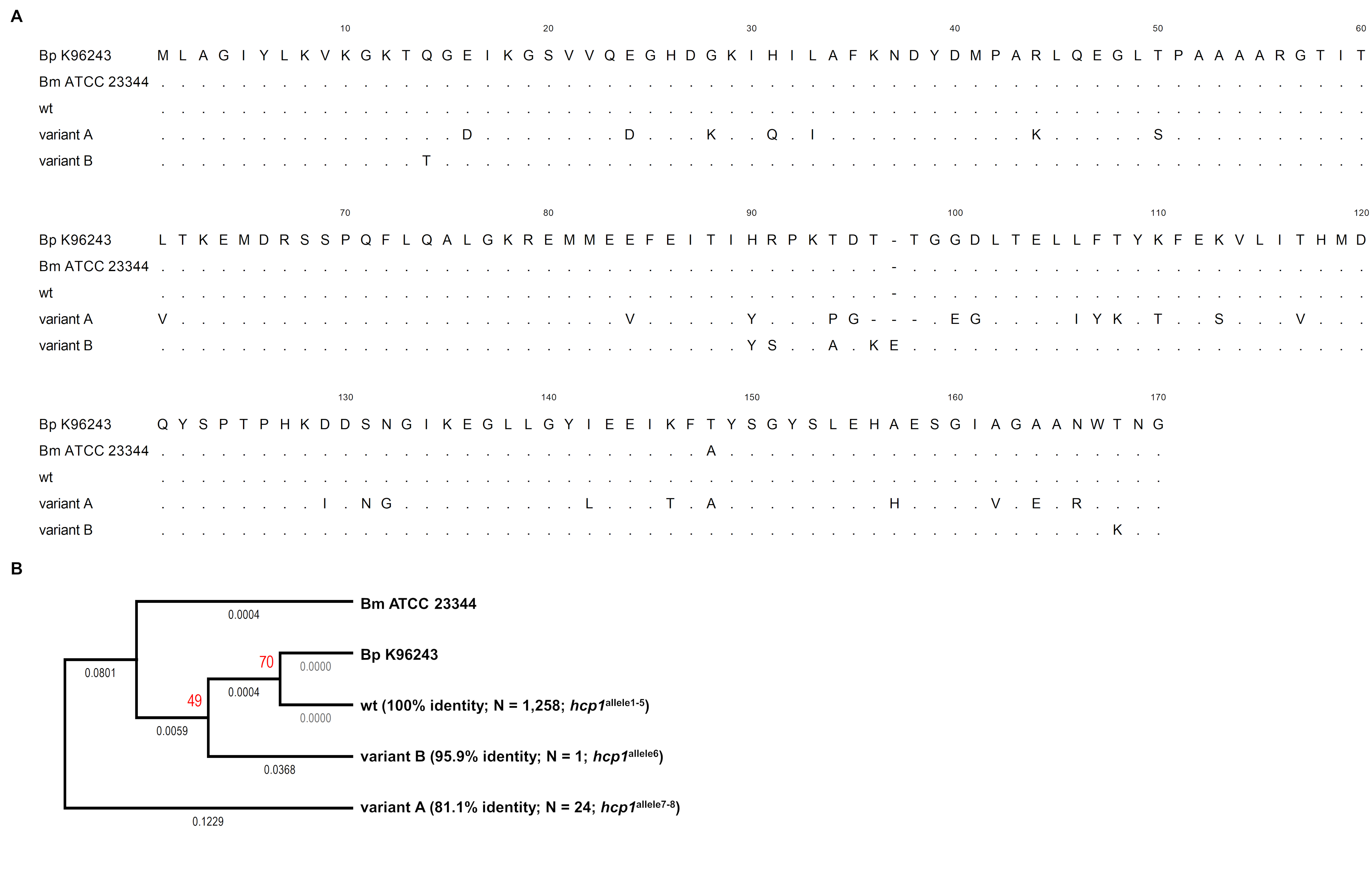

Supplement: S3 Fig — Amino acid sequence alignment (A) and phylogenetic tree based on amino acid sequences (B) of 3 Hcp1 types presented in 1,283 clinical B. pseudomallei isolates using Hcp1 of B. pseudomallei K96243 and B. mallei ATCC23344 as references. (TIF) [file pntd.0012758.s003.tif]

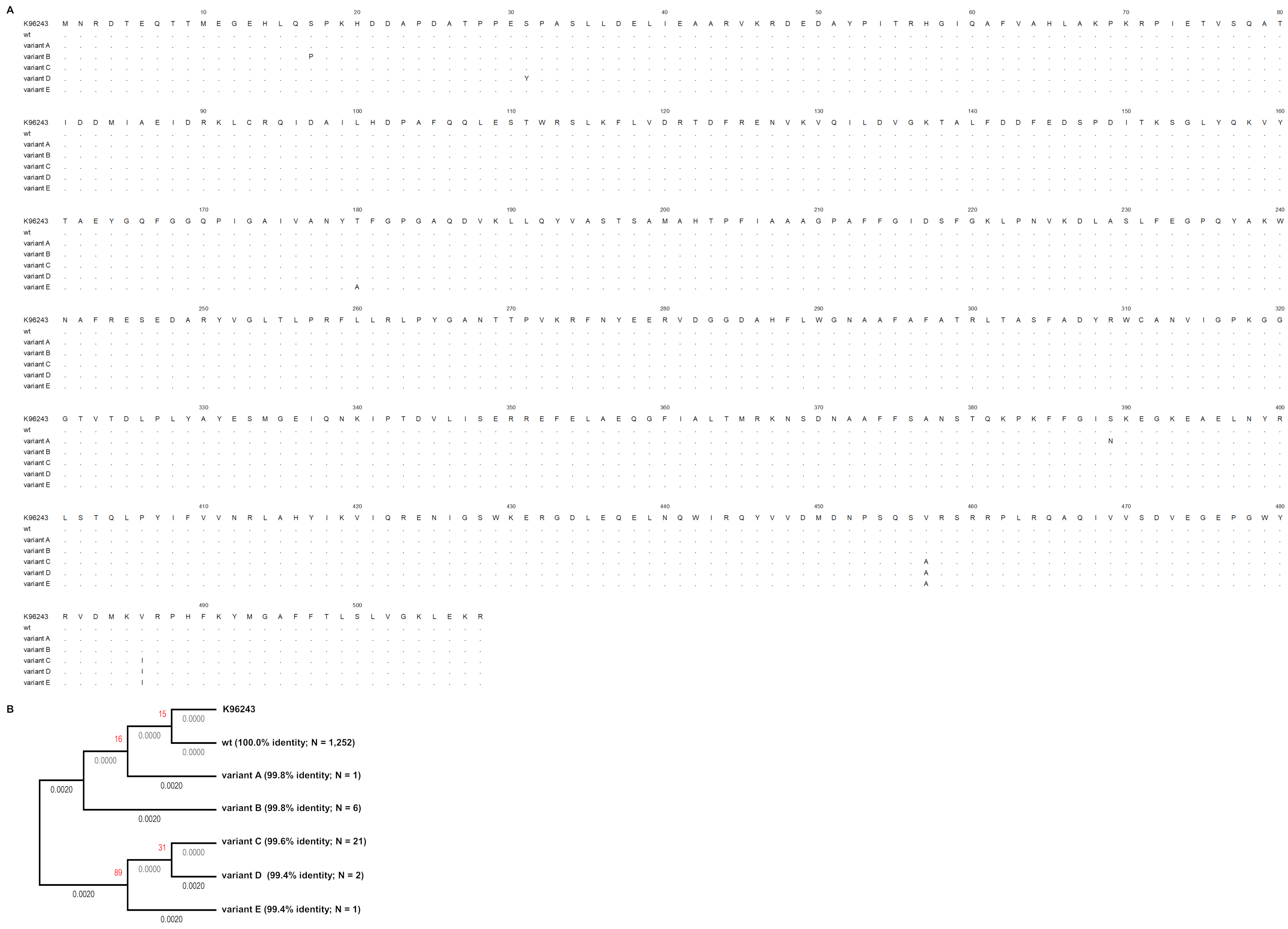

Supplement: S4 Fig — Amino acid sequence alignment (A) and phylogenetic tree based on amino acid sequences (B) of TssC B. pseudomallei K96243 (wt), and 5 TssC variants (A-E) presented in 1,283 clinical B. pseudomallei isolates. (TIF) [file pntd.0012758.s004.tif]

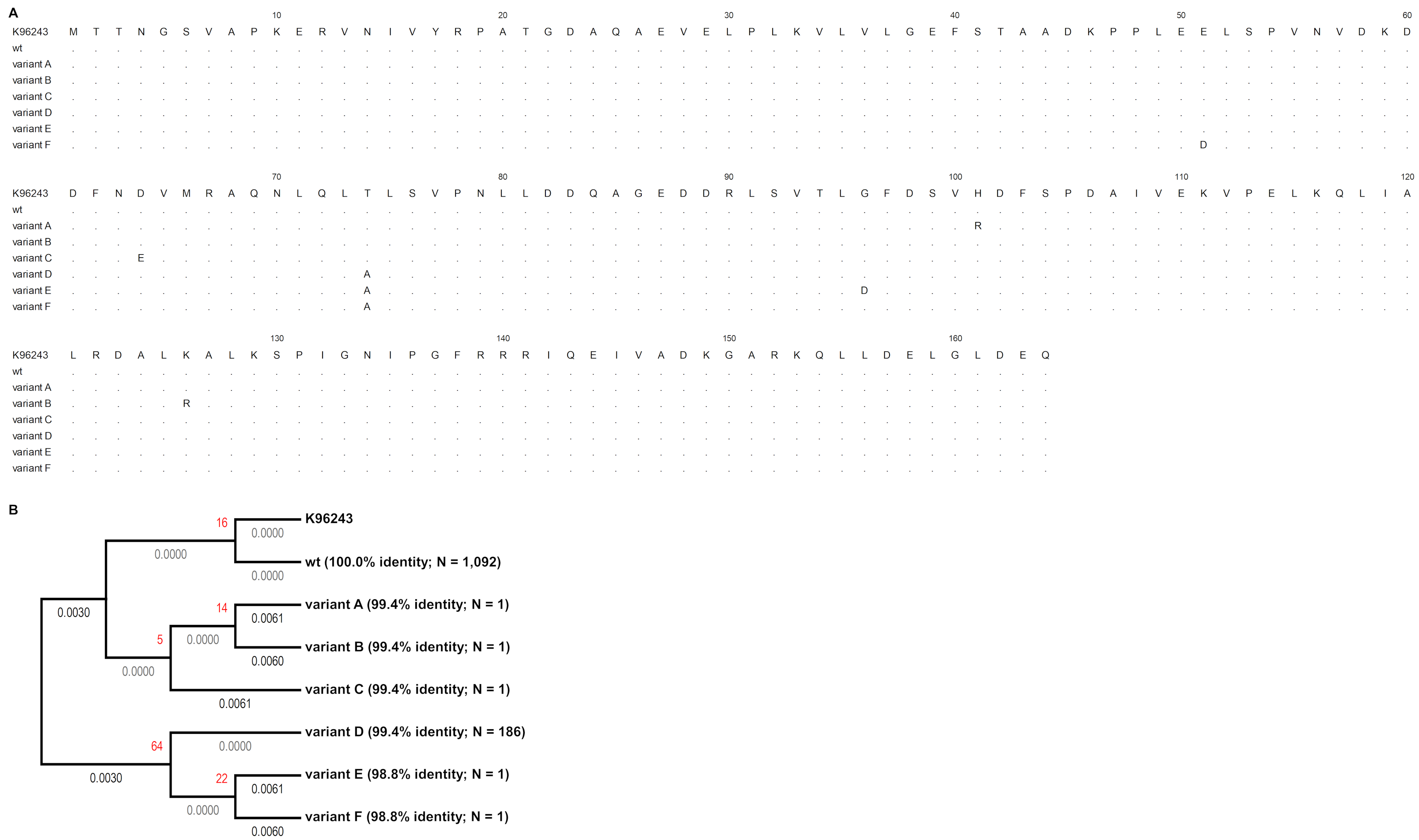

Supplement: S5 Fig — Amino acid sequence alignment (A) and phylogenetic tree based on amino acid sequences (B) of TssB B. pseudomallei K96243 (wt), and 6 TssB variants (A-F) presented in 1,283 clinical B. pseudomallei isolates. (TIF) [file pntd.0012758.s005.tif]

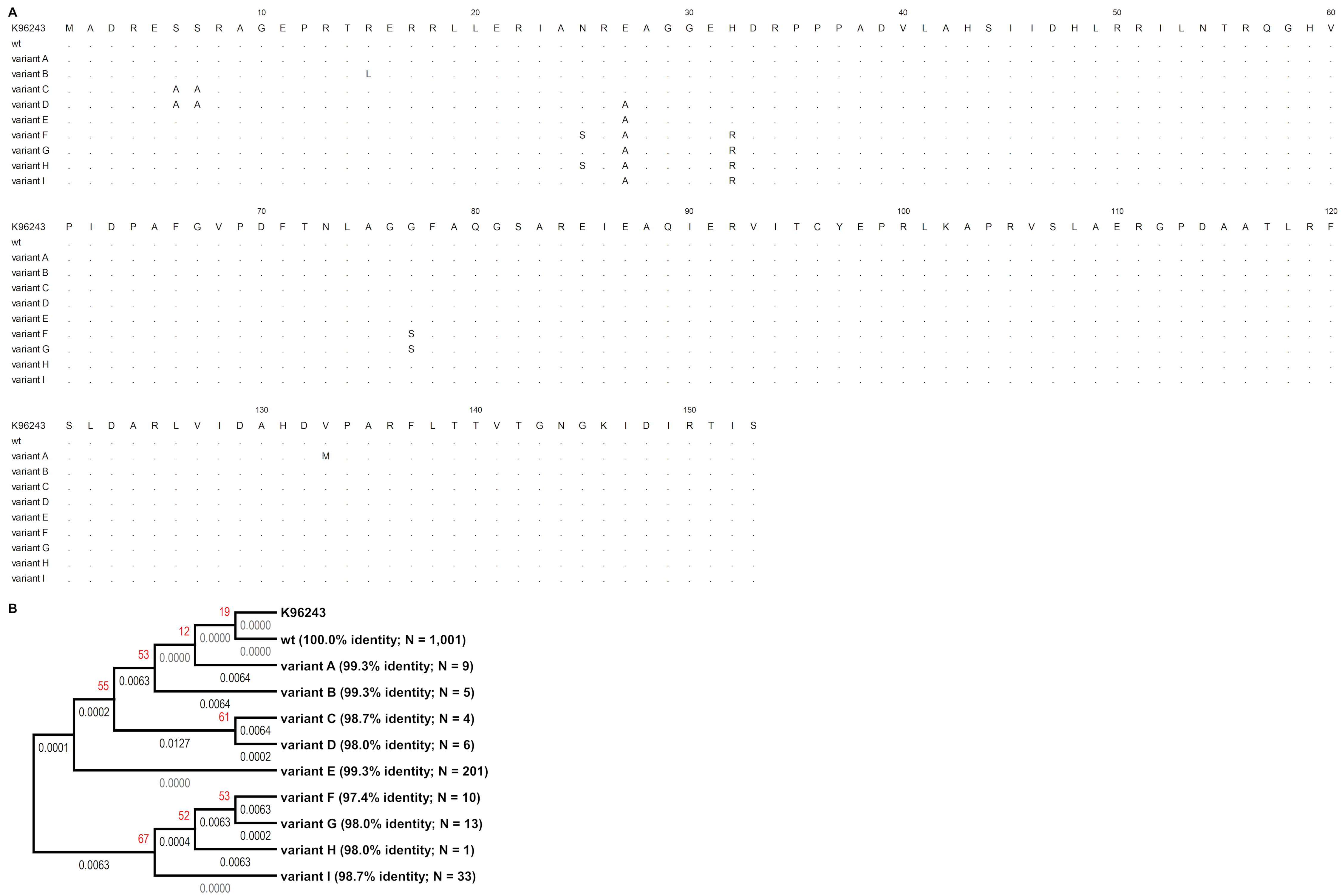

Supplement: S6 Fig — Amino acid sequence alignment (A) and phylogenetic tree based on amino acid sequences (B) of TssE B. pseudomallei K96243 (wt), and 9 TssE variants (A-I) presented in 1,283 clinical B. pseudomallei isolates. (TIF) [file pntd.0012758.s006.tif]

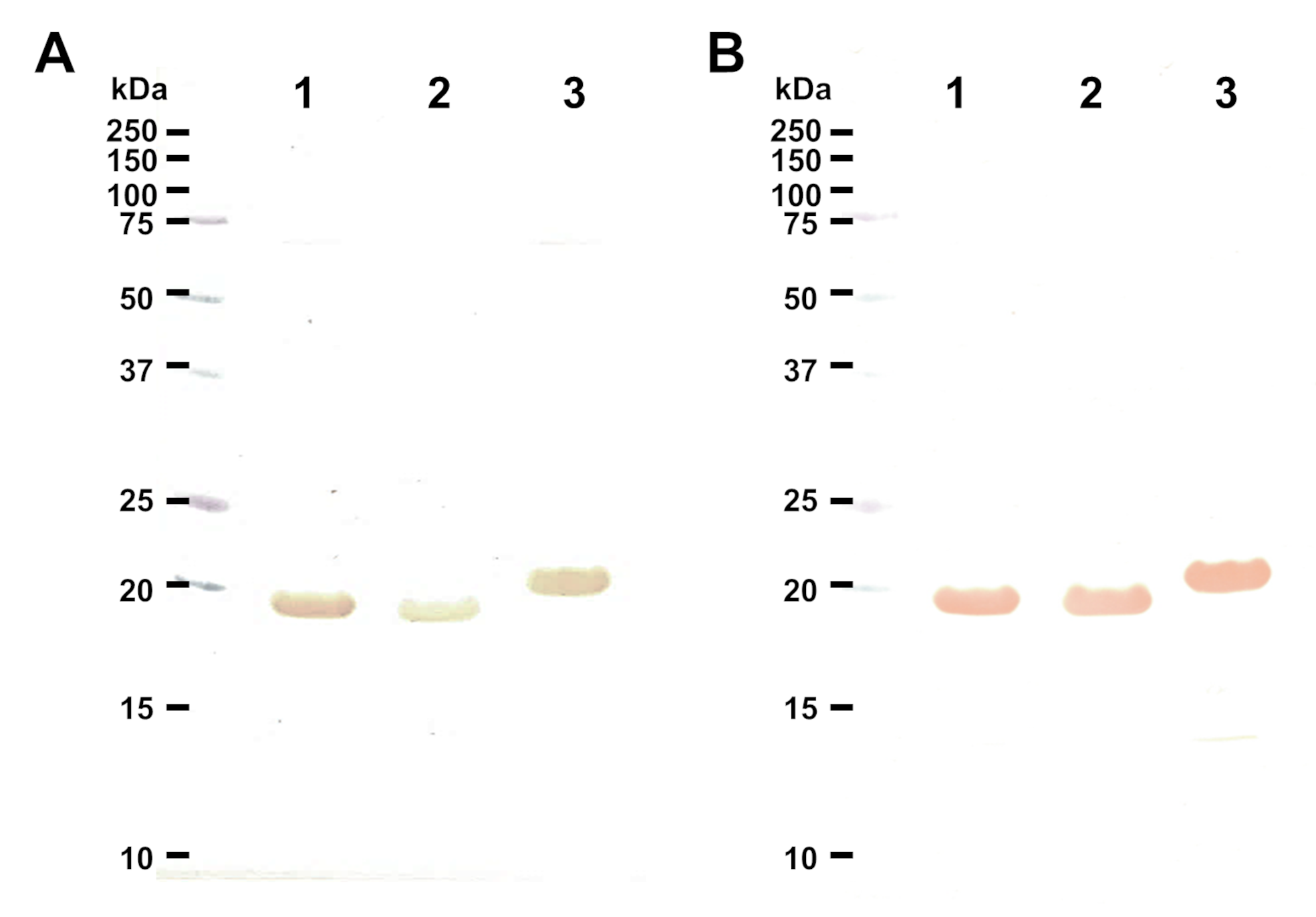

Supplement: S7 Fig — Three purified recombinant Hcp1 proteins, including rHcp1wt (Lane1), rHcp1variant A (Lane2) and rHcp1variant B (Lane3) were blotted on PVDF membrane, reacted to anti-rHcp1 (A) and anti-rHcp1variant A (B), detected with anti-mouse immunoglobulin conjugated with HRP and then visualized by 3,3’-diaminobenzidine. (TIF) [file pntd.0012758.s007.tif]

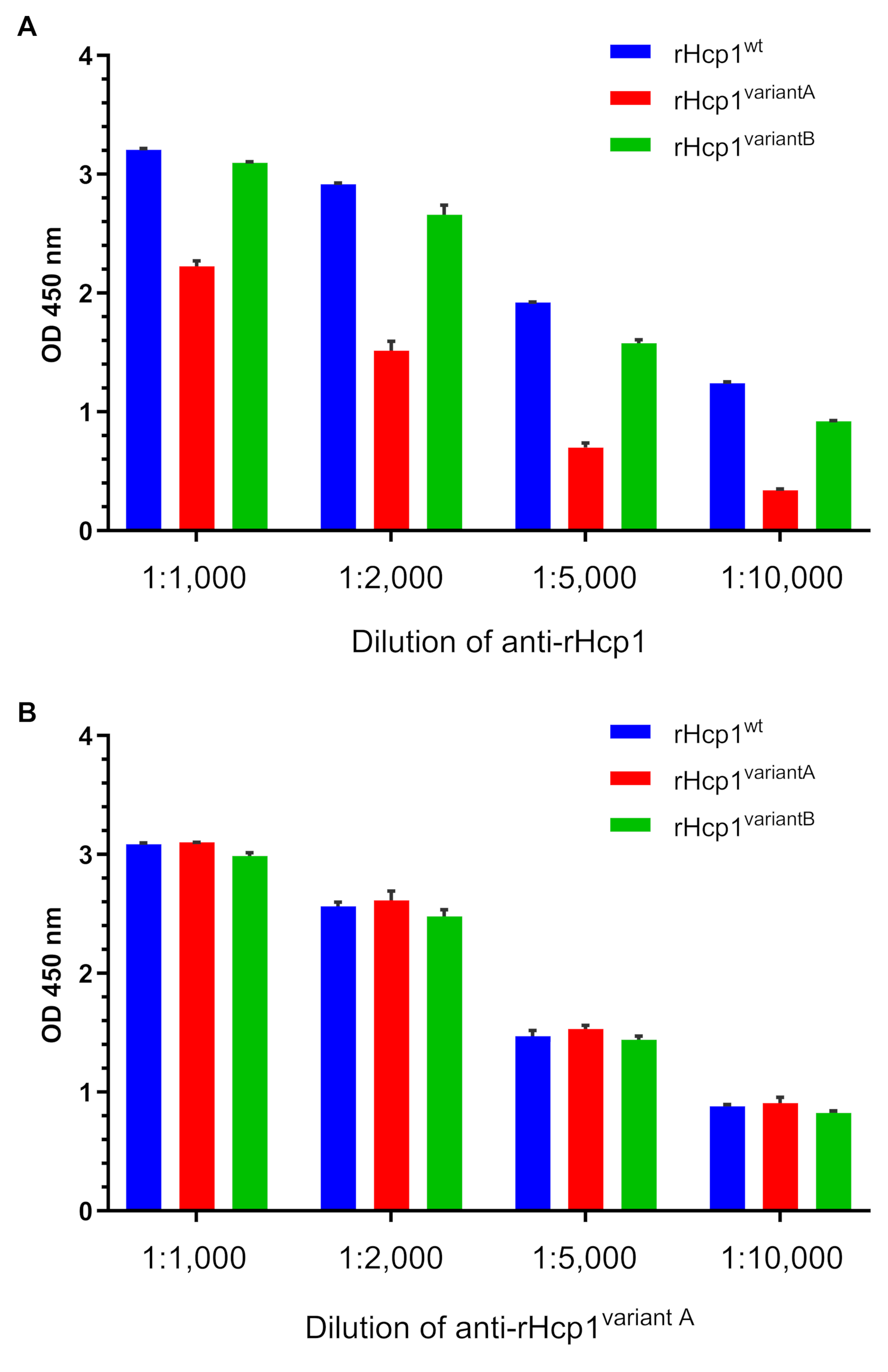

Supplement: S8 Fig — Reactivity of various dilutions of mouse sera obtained from rHcp1(A) and rHcp1variant A (B) immunizations against rHcp1wt (blue bar), rHcp1variant A (red bar) and rHcp1variant B (green bar). The experiment was performed in duplicate. The bar graph represents mean of OD values and error bar shows SD. (TIF) [file pntd.0012758.s008.tif]

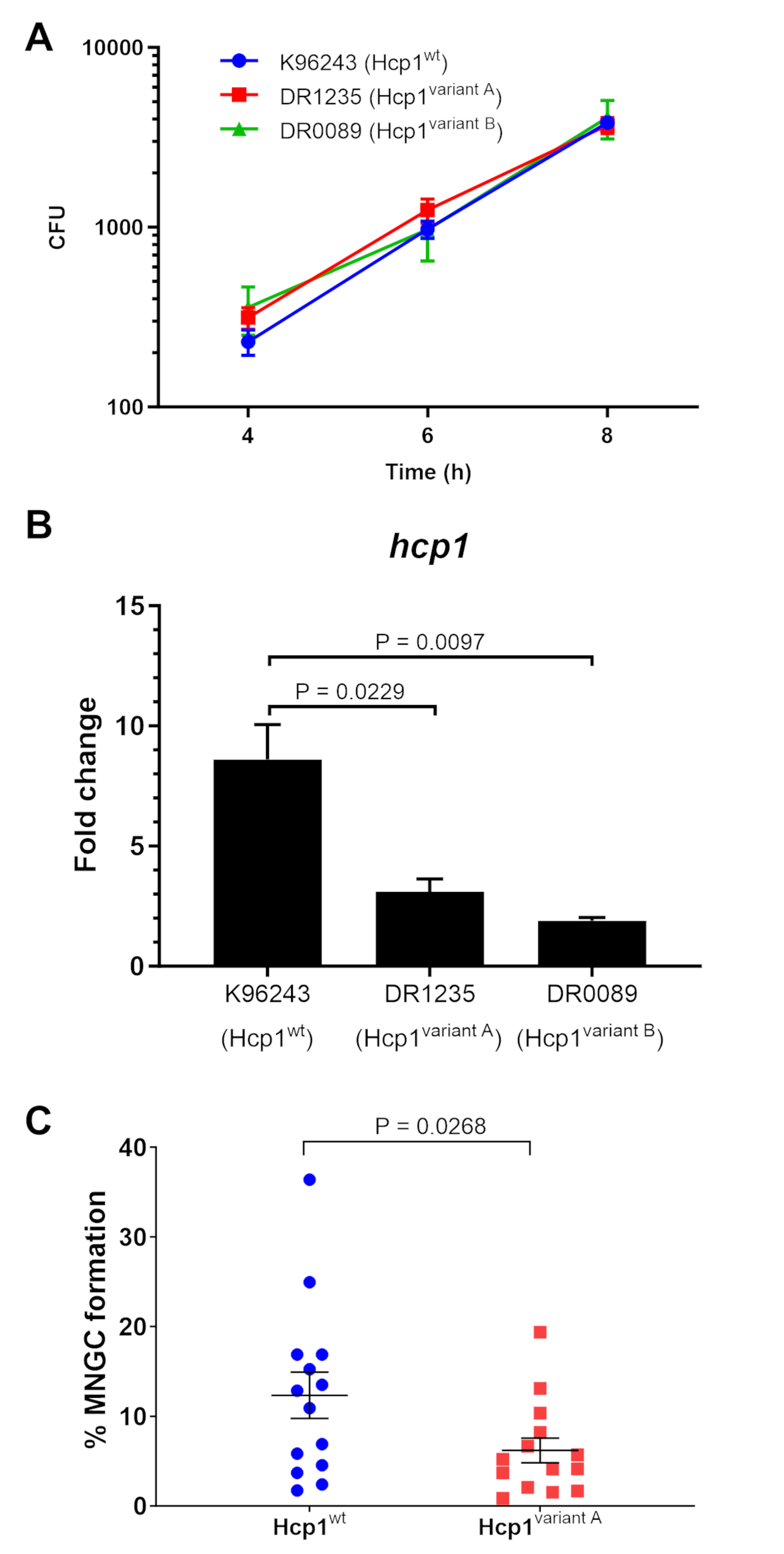

Supplement: S9 Fig — (A) Intracellular replication of B. pseudomallei in A549 cells after infection at MOI 50. (B) hcp1 gene expression by B. pseudomallei after cultured in RPMI1640 medium supplemented with 200 μM glutathione for 2 h. (C) Percentage of MNGC formation by randomly selected clinical B. pseudomallei isolates. (TIF) [file pntd.0012758.s009.tif]

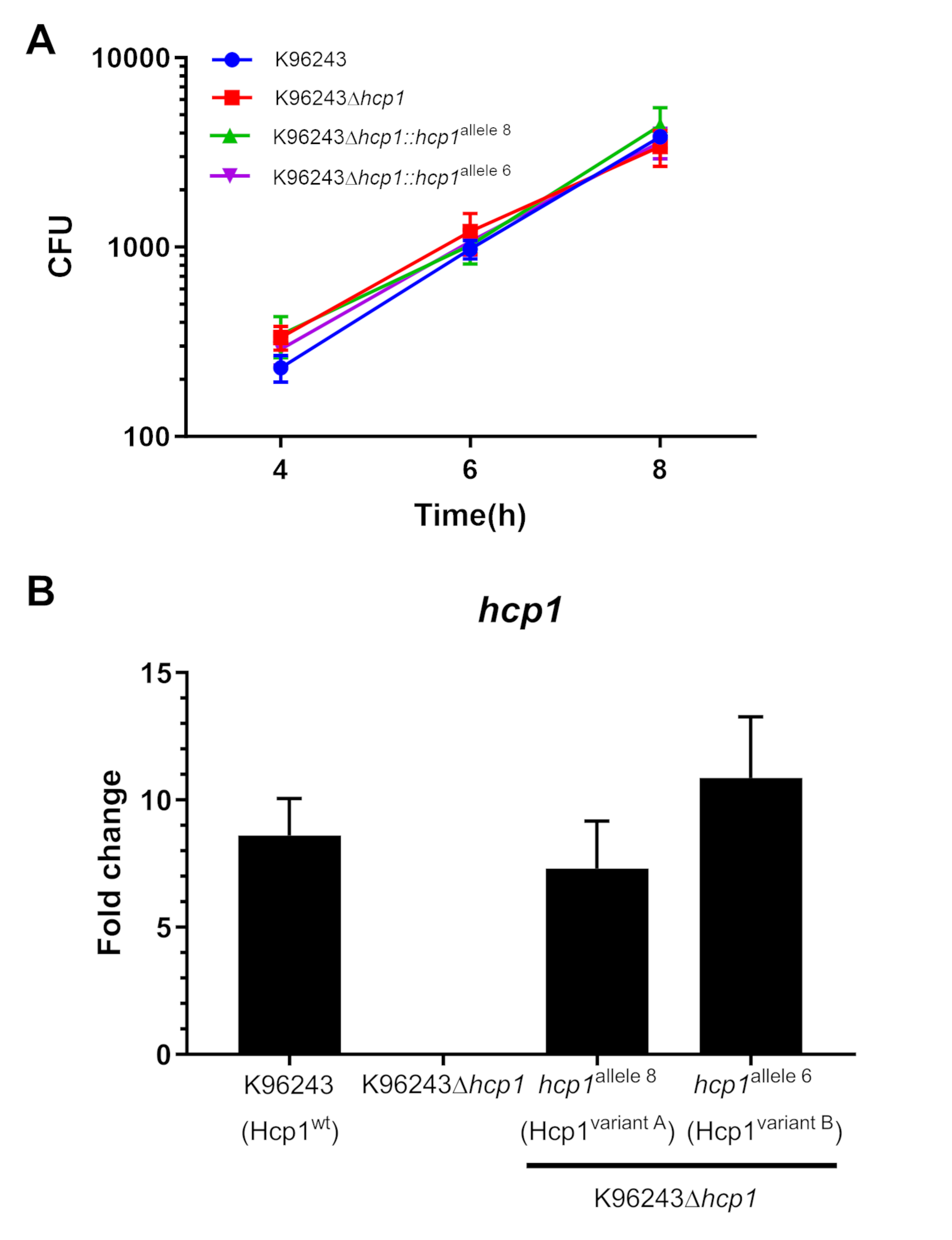

Supplement: S10 Fig — (A) Intracellular replication of B. pseudomallei in A549 cells after infection at MOI 50. (B) hcp1 gene expression by B. pseudomallei after cultured in RPMI1640 medium supplemented with 200 μM glutathione for 2 h. (TIF) [file pntd.0012758.s010.tif]
