# Supplementary material for: Genetic variation of hemolysin co-regulated protein 1 affects the immunogenicity and pathogenicity of Burkholderia pseudomallei
Source: PLoS Negl Trop Dis. 2025 Jan 6;19(1):e0012758. doi: 10.1371/journal.pntd.0012758 (PMC11737846; doi:10.1371/journal.pntd.0012758)
Supplement: S1 Text — (PDF) [file pntd.0012758.s028.pdf]

Supplementary method

Diffraction data collection and crystal structure analysis

Diffraction data sets were collected at the TPS05a beamline at National Synchrotron Radiation Research Center (NSRRC, Hsinchu, Taiwan) with a Rayonix MX300HS CCD detector, at the temperature of 100 K and the wavelength of 0.999840 Å. The diffraction data were processed using autoPROC (1). Data collection and processing statistics are shown in S3 Table.

All subsequent computations were carried out using programs from the CCP4 suite (2, 3). The initial molecular replacement solution was obtained using Phaser and the $Hcp1^{wt}$ monomer (PDB ID 3wx6; 4) as a template. The structure was refined with REFMAC alternating with manual model correction in COOT (5).

High R-factors after initial refinement and the presence of unmodelled electron density overlapping with already modelled one were indicative of a partial crystal disorder. Thorough scrutiny of the experimental data (using 3D viewers along with image viewers) showed a diffuse diffraction pattern between the layers of clearly defined diffraction spots. Figs 1-2 in S1 Text show a sample diffraction pattern, the schematic of the diffraction and the putative structure that causes such a diffraction. The ordered domain has unit cell dimensions of $a = b = 143.3$ Å, $c = 128.1$ Å. (Autoindexing based on well-defined spots gives $a_0 = b_0 = 82.7$ Å, $c_0 = 64.1$ Å). The adjacent ordered domains are offset by $\mathbf{c}/2 +/- (\mathbf{b} - \mathbf{a})/3 + n_1\mathbf{a} + n_2\mathbf{b} + n_3\mathbf{c}$, where n_1, n_2 and n_3 are integers, as shown in Fig 2B in S1 Text. Such partially disordered crystals would diffract as was observed for our crystals, with reflections $l = 2n$, $h - k = 3n$ well defined, reflections $l = 2n$, $h - k = 3n$ +/- 1 extinct (these are pseudo extinctions due to translational non-crystallographic symmetry), reflections $l = 2n + 1$, $h - k = 3$n extinct (due to destructive interference between neighbouring

ordered domains) and reflections $l = 2n + 1$, $h - k = 3n$ +/- 1 diffuse (due to small sizes of ordered crystal domains), as shown in Fig 2A in S1 Text.

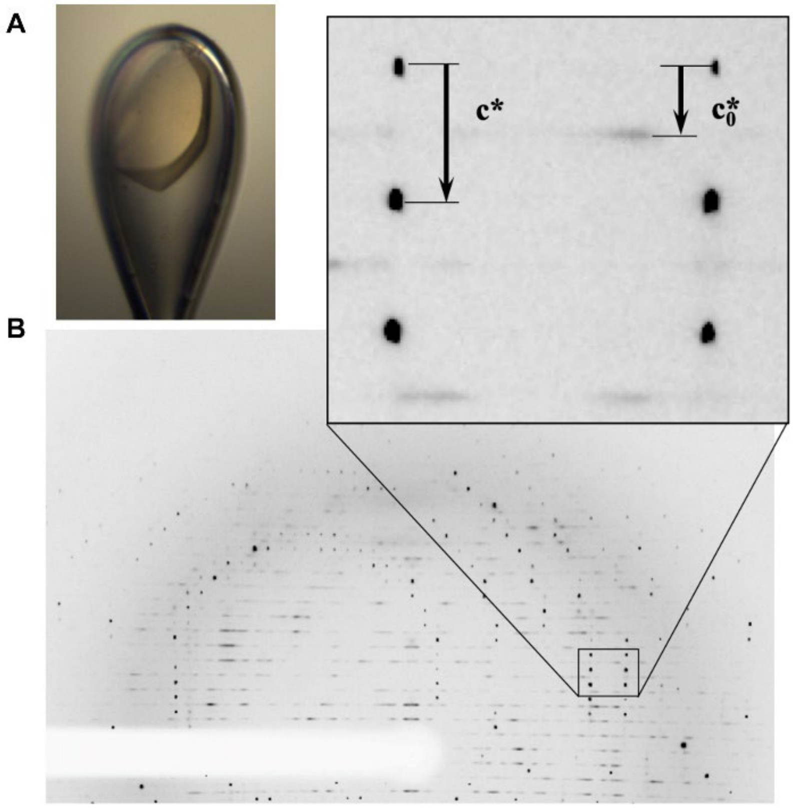

Fig 1 in S1 Text. X-ray diffraction by Hcp1$^{\text{variant B}}$ crystals. (A) Crystal of Hcp1$^{\text{variant B}}$ and (B) one of the diffraction images, where layers of properly shaped spots are clearly seen to alternate with layers of diffuse spots suggesting a partial disorder of the crystal. The direction of the diffuse patterns indicates a lack of global periodicity in the directions perpendicular to **c*** (i.e. perpendicular to the crystallographic six-fold axis), which are also the directions of faster crystal growth as can be seen from the shape of the crystal in (A). In the insert, $\mathbf{c}^*_0$ corresponds to the unit cell parameters obtained from data processing and **c*** corresponds to unit cell parameters of the ordered crystal domain.

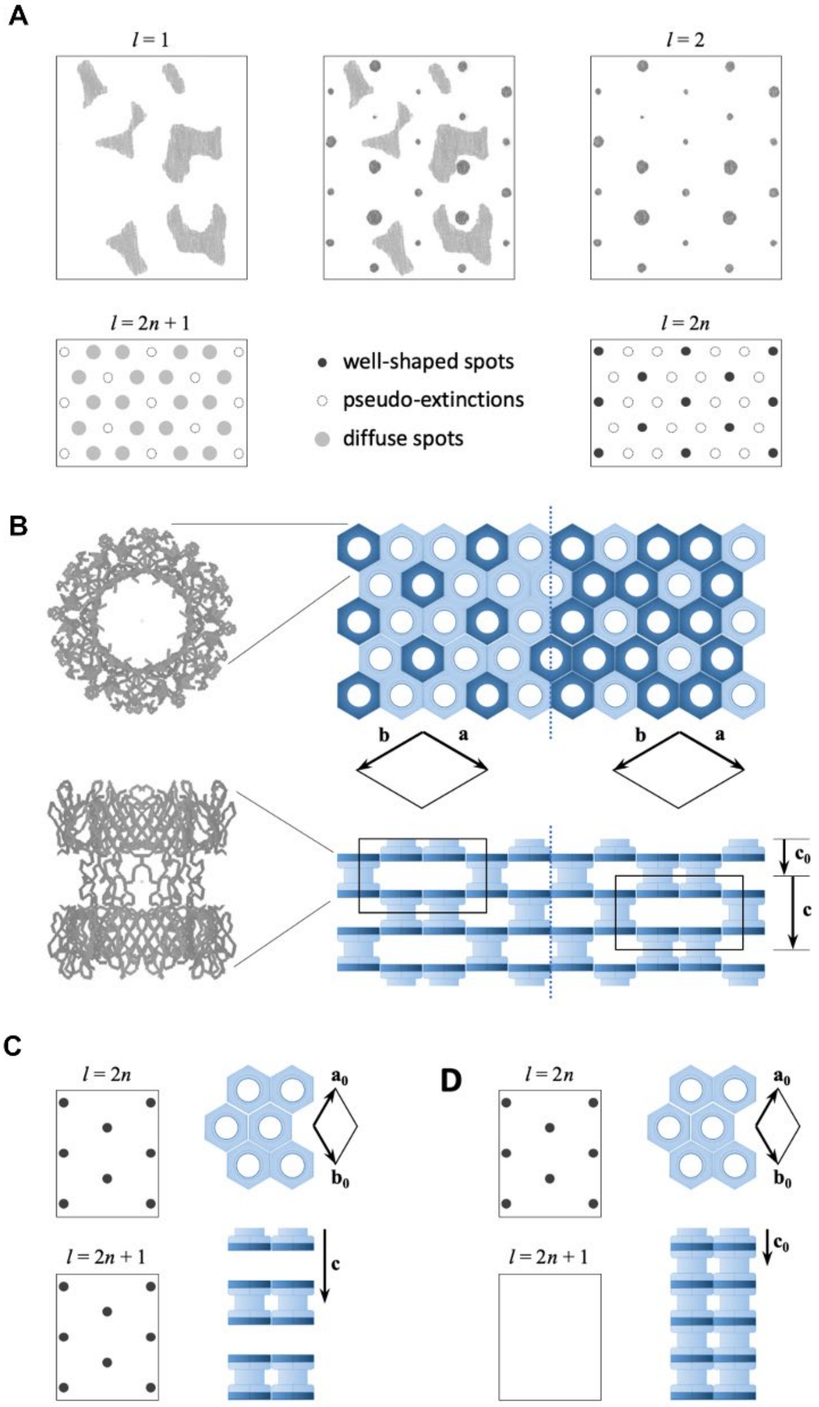

Fig 2 in S1 Text. Partial disorder in Hcp1$^{\text{variant B}}$ crystal. (A) Three slices of the crystal diffraction presented in 3-D using dials.rs_mapper and COOT, and their interpretation in terms of well-defined, extinct and diffuse reflections. (B) Two adjacent ordered crystal domains of a putative

Hcp1 crystal structure, which would diffract as in (A), with reflections $l = 2n$, $h - k = 3n$ well defined, reflections $l = 2n$, $h - k = 3n$ +/- 1 extinct due to translational non-crystallographic symmetry, reflections $l = 2n + 1$, $h - k = 3n$ extinct due to destructive interference between neighbouring ordered crystal domains, and reflections $l = 2n + 1$, $h - k = 3n$ +/- 1 diffuse due to small sizes of these domains. (C, D) Periodic representations of (A), which can be used in refinement. (C) implies refinement against 50% complete data and allows partial deconvolution of electron density maps by replacing unmeasured Fobs of the reflection $l = 2n + 1$ with Fcalc. (D) implies refinement against 100% data, but molecules in the refined structure overlap with symmetry-related molecules.

Figs 2C-D in S1 Text show two different ways of representing our disordered non-periodic crystal as a periodic structure in the sense that calculated structure amplitudes of the representation match the well-defined fraction of reflections, whose intensities were measured. The representation shown in Fig 2D in S1 Text, with $a_0 = b_0 = 82.7$ Å, $c_0 = 64.1$ Å and molecules overlapping with their symmetry mates, has been deposited with the PDB (PDB ID 8Z7K).

In contrast to the model of the crystal structure proposed in (4), where hexamers were suggested to form dodecamers with, in effect, physically impossible contacts and further on, to connect into continuous tubes through the loops 41-55, as in Fig 2D in S1 Text, the hexamers in our model in Fig 2B in S1 Text do not form continuous tubes. They are connected through loops 41-55 to form non-biological dodecamers, which are separated by big gaps in the direction of the six-fold axis giving sufficient space for loops 92-103 and 124-140 disordered and invisible in both 3wx6 and our structures. In the ordered domains, the dodecamers sitting on the crystallographic six-fold axes are shifted by **c**/2 relative to the hexamers sitting on the crystallographic three-fold

axes to bridge discontinuous columns of hexamers residing on each axis and to make the crystal structure connected in 3-D. Later comparisons with homologous structures from the PDB revealed that the crystal structure of Hcp3 from *P. aeruginosa* (PDB ID 3he1; 6) is very similar, in its overall organisation, to a putative ordered domain in our structure shown in Fig 2B in S1 Text.

Given that the space group in the PDB entry 3wx6 was the same as in the small-cell representation of our structure, Fig 2D in S1 Text, parameters *a* and *b* differed by less than 0.02 Å, and *c* differed by only 0.75 Å, the crystal used in 3wx6 was very likely to have the same pathology as our crystals. We downloaded experimental data 3wx6 and re-refined our new structure against these data to $R = 0.214$ and $R_{\mathrm{free}} = 0.267$. (For comparison, $R = 0.249$ and $R_{\mathrm{free}} = 0.285$ were reported for 3wx6 after TLS refinement with a twin refinement target, which usually reduces R-factors even if the crystal was not a twin). We concluded that we deal with isomorphous crystal forms in 3wx6 and our cases.

Monomer A from the Hcp1$^{\mathrm{variant\ B}}$ 1.58 Å structure was superposed on two contacting monomers from the adjacent hexamers of the T6SS sheath/tube complex in *V. cholerae* (PDB ID 5ojq; 7) using the SSM superpose tool in COOT to analyze the effect of mutations.

References

1. Vonrhein C, Flensburg C, Keller P, Sharff A, Smart O, Paciorek W, et al. Data processing and analysis with the autoPROC toolbox. Acta Crystallogr D Biol Crystallogr. 2011;67(Pt 4):293-302.
2. Winn MD, Ballard CC, Cowtan KD, Dodson EJ, Emsley P, Evans PR, et al. Overview of the CCP4 suite and current developments. Acta Crystallogr D Biol Crystallogr. 2011;67(Pt 4):235-42.

3. Krissinel E, Lebedev AA, Uski V, Ballard CB, Keegan RM, Kovalevskiy O, et al. CCP4 Cloud for structure determination and project management in macromolecular crystallography. Acta Crystallogr D Biol Crystallogr. 2022;78(Pt 9):1079-89.
4. Lim YT, Jobichen C, Wong J, Limmathurotsakul D, Li S, Chen Y, et al. Extended loop region of Hcp1 is critical for the assembly and function of type VI secretion system in *Burkholderia pseudomallei*. Sci Rep. 2015;5:8235.
5. Emsley P, Lohkamp B, Scott WG, Cowtan K. Features and development of Coot. Acta Crystallogr D Biol Crystallogr. 2010;66(Pt 4):486-501.
6. Mougous JD, Cuff ME, Raunser S, Shen A, Zhou M, Gifford CA, et al. A virulence locus of *Pseudomonas aeruginosa* encodes a protein secretion apparatus. Science. 2006;312(5779):1526-30
7. Wang J, Brackmann M, Castano-Diez D, Kudryashev M, Goldie KN, Maier T, et al. Cryo-EM structure of the extended type VI secretion system sheath-tube complex. Nat Microbiol. 2017;2(11):1507-12.
